# Sustained Liver HBsAg Loss and Clonal T- and B-Cell Expansion upon Therapeutic DNA Vaccination Require Low HBsAg Levels

**DOI:** 10.3390/vaccines11121825

**Published:** 2023-12-06

**Authors:** Nádia Conceição-Neto, Wim Pierson, Maurizio Vacca, Matthias Beyens, Ben De Clerck, Liese Aerts, Birgit Voeten, Dorien De Pooter, Lore Verschueren, Koen Dockx, Mathias Vandenberk, Ewoud De Troyer, Kato Verwilt, Carl Van Hove, Mieke Verslegers, Leslie Bosseler, Marjolein Crabbe, Vinod Krishna, Isabel Nájera, Ellen Van Gulck

**Affiliations:** 1 Infectious Diseases Discovery, Infectious Diseases and Vaccines, Janssen Research and Development, Turnhoutseweg 30, 2340 Beerse, Belgium; nneto@its.jnj.com (N.C.-N.); mvacca@its.jnj.com (M.V.); bdeclerc@its.jnj.com (B.D.C.); lieseaerts@hotmail.com (L.A.); ddepoote@its.jnj.com (D.D.P.); lversch2@its.jnj.com (L.V.); 2 Discovery Therapeutics and Molecular Pharmacology, Janssen Research and Development, Turnhoutseweg 30, 2340 Beerse, Belgium; mbeyens@its.jnj.com (M.B.); kverwilt@its.jnj.com (K.V.); cvhove@its.jnj.com (C.V.H.); 3 Charles River Laboratories, Turnhoutseweg 30, 2340 Beerse, Belgiumkdockx@its.jnj.com (K.D.); mvanden6@its.jnj.com (M.V.); 4 SDS Discovery Statistics, Janssen Research and Development, Turnhoutseweg 30, 2340 Beerse, Belgium; edetroye@its.jnj.com (E.D.T.); mcrabbe1@its.jnj.com (M.C.); 5 Preclinical Sciences and Translational Safety (PSTS) Janssen Research and Development, Turnhoutseweg 30, 2340 Beerse, Belgium; mversleg@its.jnj.com (M.V.); lbossele@its.jnj.com (L.B.); 6 Infectious Diseases Discovery, Infectious Diseases and Vaccines, Janssen Research and Development, 1400 McKean Road, Spring House, PA 19002, USA; vkrish10@its.jnj.com; 7 Infectious Diseases and Vaccines, Janssen Research and Development, 1600 Sierra Point Parkway, South San Francisco, CA 94005, USA; inajera@its.jnj.com

**Keywords:** HBV, single-cell-RNA sequencing, liver, cytotoxic CD8 T cells, CD4 follicular helper T cells, vaccine

## Abstract

Background: Suppression of HBV DNA, inhibition of HBV surface (HBsAg) production and therapeutic vaccination to reverse HBV-specific T-cell exhaustion in chronic HBV patients are likely required to achieve a functional cure. In the AAV-HBV mouse model, therapeutic vaccination can be effective in clearing HBV when HBsAg levels are low. Using a single-cell approach, we investigated the liver immune environment with different levels of HBsAg and sustained HBsAg loss through treatment with a GalNAc-HBV-siRNA followed by therapeutic vaccination. Methods: AAV-HBV-transduced C57BL/6 mice were treated with GalNAc-HBV-siRNA to lower HBsAg levels and then vaccinated using a DNA vaccine. We used single-cell RNA and V(D)J sequencing to understand liver immune microenvironment changes. Results: GalNAc-HBV-siRNA, followed by therapeutic vaccination, achieved sustained HBsAg loss in all mice. This was accompanied by CD4 follicular helper T-cell induction, polyclonal activation of CD8 T cells and clonal expansion of plasma cells that were responsible for antibody production. Conclusions: This study provides novel insights into liver immune changes at the single-cell level, highlighting the correlation between induced reduction of HBsAg levels and clonal expansion of CD4, CD8 T cells and plasma cells in the liver upon HBV siRNA and subsequent therapeutic vaccination.

## 1. Introduction

The hepatitis B virus (HBV) poses a major global health problem, with the World Health Organization (WHO) estimating 1.5 million new infections yearly and nearly 296 million people living with chronic hepatitis B (CHB) infection, which is often life-long [1]. Approximately 20–30% of individuals with CHB develop cirrhosis, liver failure or hepatocellular carcinoma [1]. CHB has various clinical stages defined by HBV DNA levels, the presence or absence of hepatitis B e antigen (HBeAg) and the presence or absence of liver inflammation [1].

The current standard of care is antiviral nucleos(t)ide analogue (NUC) therapy, which suppresses viral replication by inhibiting reverse transcription but does not target the nuclear covalently closed circular DNA (cccDNA), therefore resulting in the persistence of HBV [2]. Given that viral replication is suppressed, and it largely eliminates the risk of liver cirrhosis but only partially the risk of hepatocellular carcinoma, it tends to be a lifelong therapy since, upon withdrawal, the viral load usually rebounds, driven by cccDNA [3]. For the subset of patients who can stop NUC therapy (HBeAg-, undetectable HBV DNA and low HBsAg), <3% achieve functional cure through an unknown mechanism of action but with the key contribution of the HBV-specific adaptive immune response as observed in those who recover from acute infection. Used less often than NUC is finite treatment (typically 48 weeks) with pegylated IFN-alpha (+/−NUC), which can induce loss of HBV surface antigen (HBsAg) in up to 10% of patients [4,5]. However, its low tolerability due to the well-known side effects of IFN-based treatments can lead to early treatment discontinuation [6].

The current goal in HBV therapy is to achieve a functional cure, defined as sustained loss of HBsAg (with or without HBsAg seroconversion) and undetectable HBV DNA in serum 24 weeks off treatment [7], where a functional HBV-specific adaptive immune response can control the infection [6]. How HBV establishes a chronic infection in those that progress to CHB remains unclear, but in acute HBV infection, HBV-specific CD4, CD8 T cells and neutralizing antibodies are key to limit the infection. Spontaneous immune control has been described in around 0.5% of chronically infected patients and is also accompanied by neutralizing antibodies and HBV-specific CD4 and CD8 T cells [8]. However, HBV persistence is characterized by a lack of neutralizing antibodies, dysfunctional, exhausted HBV-specific T and B cells, and inhibition and exhaustion of natural killer (NK) cells [9,10]. Although there is no definitive evidence of the role of HBsAg in T-cell dysfunction or exhaustion, a current hypothesis is that the reduction of HBsAg levels is not sufficient but necessary to facilitate or mediate the gain in T-cell function to reach a functional cure [11].

High levels of HBsAg (due to overproduction by a factor of 4 to 5 log_10_ of subviral particles over infectious Dane particles) [12] are believed to be one of the mechanisms contributing to the sustained suppression of HBV-specific immune responses, which hampers therapeutic vaccination [13]. This hypothesis is also supported by the observation in animal models of CHB, which show that therapeutic vaccination is more effective when HBsAg levels are low (below 100 IU/mL), and it is not effective when HBsAg levels are >1000 IU/mL [14]. In clinical setting studies, CHB-infected individuals that received VPT-300, a prime-boost regimen whereby the immune system is primed with an adenovirus (ChAdOx1) and boosted with a pox virus (MVA), showed significant and durable reductions of HbsAg, but only in those patients with baseline HBsAg levels <50 IU/mL [15].

To better understand the liver immune microenvironment in chronically HBV-infected patients, fine needle aspirates from patients at different stages of the disease have been profiled using single-cell RNA sequencing [16,17,18,19]. Nkongolo et al. profiled the T-cell response in patients undergoing antiviral therapy in-depth and observed that CD8 hepatotoxic T cells led to nonspecific hepatocyte killing, leading to fibrosis and cirrhosis [17].

In this paper, we aimed to further understand the role of HBV antigenemia (i.e., HBs- and HBeAg levels) on the liver immune environment to define key CD4 and CD8 T-cell populations and their ability to control the infection upon vaccination. Single-cell RNA, T-cell receptor (TCR) and B-cell receptor (BCR) variability, diversity, and joining (V(D)J) sequencing of liver resident cells from (un)treated AAV-HBV infected mice were performed. We provide important insights into the changes in the liver microenvironment upon and during vaccination and in the context of different HBV levels.

## 2. Materials and Methods

### 2.1. Compounds

Therapeutic vaccine consisted of pDF-core, pDF-pol [20] and pDK-s plasmids.

GalNAc-conjugated HBV siRNA (GalNAc-HBV siRNA) and GalNAc-conjugated control siRNA (GalNAc-control siRNA) were manufactured by Arrowhead Pharmaceuticals (Passadena, CA, USA) and Axolabs (Kulmbach, Germany, respectively, for research purposes only.

### 2.2. Animal Models and Study Designs

**Figure 1:**
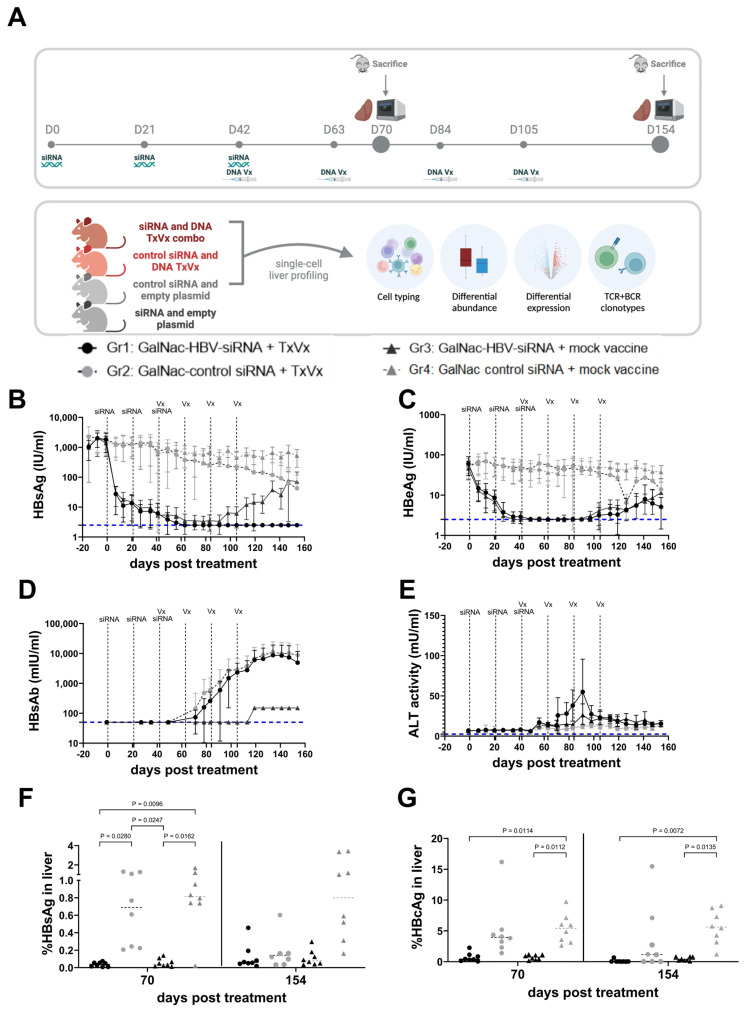
Effect on HBV viral parameters by sequential treatment of siRNA and therapeutic vaccination in AAV-HBV mice. Female C57/Bl6 were transduced with 2.5 × 109 vge of AAV-HBV (**A**) 4 weeks after transduction; treatment started with siRNA and was followed by therapeutic vaccine encoding Core, Pol and surface antigens. Mice were vaccinated every 3 weeks (indicated with dotted vertical lines and Vx) for 4 times after 3 doses of siRNA (indicated with dotted vertical lines and siRNA) (figure made with Biorender). Every week, blood was taken from each mouse, and serum was collected for measurement of HBsAg (**B**), HBeAg (**C**), HBsAb (**D**) and ALT (**E**) over time. Eight mice were sampled at day 70 (1 week after second vaccination); the other mice were sampled at day 154 (7 weeks after last vaccination). Immunohistochemistry on FFPE livers was used to semi-quantitatively assess HBsAg (**F**) and HB core Ag (**G**). Mixed effect analysis and Tukey’s multiple comparison testing were performed as statistical testing (*p* values are indicated; otherwise, it is not significant). Black circles: GalNAc-HBV siRNA and TxVx; black triangles: GalNAc-HBV siRNA and mock vaccine; grey circle: GalNAc-control siRNA and TxVx; grey triangles: GalNAc-control siRNA and mock vaccine. Lower limit of quantification (LLOQ) of each assay is indicated with horizontal blue dotted line.

Female C57BL/6 mice were transduced with 2.5 × 10^9^ vge per mouse of rAAV-HBV1.3-mer WT replicon to reach HBsAg levels of 3 log_10_ IU/mL. At day 28 after transduction, mice were treated subcutaneously with 3 mg/kg GalNAc-HBV siRNA (n = 32) or with control GalNAc-siRNA (n = 32) 3 times, between each dose were 3 weeks. At the third siRNA dose, mice were also electroporated (TriGrid electroporation system, Ichor Medical Systems, San Diego, CA, USA) with HBV vaccine containing 10µg of each plasmid (pDF-core, pDF-pol, pDK-s, 30 µg in total, n = 16) or with 30µg of empty plasmid (referred to as mock, n = 16). The vaccine was subsequently dosed 4 times every 3 weeks (Figure 1A). HBV-specific T cells were measured 1 week after the second vaccination (n = 8/group) and 7 weeks after the last vaccination (n = 8/group). Blood for viral parameters was collected every week, from which serum was prepared and stored at −80 °C until assayed. At the end of the study, the liver was formalin-fixed and embedded in paraffin to determine HBs and HB core antigen levels by immunohistochemistry. Liver resident T cells were also collected to perform single-cell RNA sequencing and V(D)J.

### 2.3. Isolation of Intrahepatic Lymphocytes (IHLs)

IHLs were obtained by perfusing the liver with PBS via the hepatic portal vein to flush out excess blood. Ex vivo perfusion, enzymatic digestion and tissue dissociation of the left lateral liver lobe were performed using a GentleMACS Octo Dissociator with heaters and the liver perfusion kit for mice according to the manufacturer’s protocol (Miltenyi Biotec, Bergisch Gladbach, Germany). Hepatocytes were separated from lymphocytes by centrifugation at 50× *g* for 5 min. Supernatant was spun down at 400× *g* for 5 min, followed by resuspension in a 33.75% (*v*/*v*) Percoll (GE Healthcare, Machelen, Belgium) diluted in PBS with 2% fetal calf serum and density gradient centrifugation at 700× *g* for 12 min. Next, residual hepatocytes and debris were discarded, and red blood cells co-sedimented with the intrahepatic immune cells (IHICs) were lysed using ACK lysis buffer (Lonza, Basel, Switserland) for 5 min. Cells were washed twice and counted. Cell concentration and viability were determined using a Nexcelom Cellaca MX Cell Counter (Lawrence, MA, USA). Splenocytes were tested at 200,000 cells/well, and IHLs were tested at 80,000 cells/well on IFN-γ ELISpot plates.

### 2.4. Detection of HBV-Specific T Cells by ELISPOT

All cells were stimulated with overlapping peptide pools covering the entire Core, Pol and S sequences (JPT, Gladbach, Germany) on pre-coated ELISpot plates (Mabtech, Nacka Strand, Sweden) to measure the number of cells that secrete IFN-γ. As a negative control, cells were stimulated with dimethyl sulfoxide (DMSO). After overnight stimulation, plates were developed following the manufacturers’ instructions (Mabtech, Sweden). The number of spot-forming cells (SFC) was counted using an ELISpot reader (CTL, Cleveland, OH, USA). The DMSO (mock control) background was subtracted from all responses. Immunogenicity results are presented as the number of SFC per million of IHL. Results are shown as mean ± standard deviation. Due to the large size of the Pol protein, peptides were split into two peptide pools, Pol1 and Pol2.

### 2.5. Viral Parameters and Alanine Aminotransferase (ALT) Analyses

Serum HBsAg, HBeAg and anti-HB levels were quantified using CLIA kits (Ig Biotechnology, Burlingame, CA, USA). All CLIA kits were used according to the manufacturer’s guidance. Depending on the estimated levels, different dilutions of serum in PBS were used. Read-out of plates was performed with a Viewlux ultra HTS microplate imager (Perkin Elmer, Mechelen, Belgium). The serum alanine aminotransferase (ALT) activity was analysed using a commercially available kit according to the manufacturer’s guidance (Sigma-Aldrich, Saint Louis, MO, USA) and a Spark multimode microplate reader (Tecan, Mechelen, Belgium). Four µL of serum were used to perform the assay.

### 2.6. Histology and Immunohistochemistry (IHC)

Formalin-fixed paraffin-embedded livers were sectioned at 5 µm thickness, mounted on pre-charged slides and stained with HBcAg (B0586, Dako, Glostrup, Denmark) and HBsAg (ab859, Abcam, Cambridge, UK). Tissues were subjected to an EDTA-based antigen retrieval while signal amplification and detection were achieved with a hapten multimer and a DAB chromogenic detection kit on the Ventana Discovery Ultra autostainer (Roche Diagnostics, Rotkreuz, Switzerland). Negative control staining was performed for every antibody using an isotype control (Rabbit PE IgG for HBcAg, mouse IgG1 for HBsAg). Sections were coverslipped with the automated Ventana HE600 (Roche Diagnostics, Rotkreuz, Switzerland) and scanned using the Hamamatsu Nanozoomer RX.

### 2.7. Image Analysis

IHC stainings were evaluated using the HALO^TM^ system (Halo 3.4). Liver tissue was discriminated from glass/lumen by using a tailored random forest classifier in HALO v3.4., and expression of HBcAg and HBsAg were evaluated using two tailored algorithms based on nuclear or signal area detection, respectively. Output parameters include percentage of HBcAg-positive cells relative to total number of haematoxylin-positive cells and HBsAg-positive surface area relative to the total classified liver area analyzed.

### 2.8. Single-Cell RNA Sequencing and Data Analysis

IHLs were processed according to 10x Chromium Single-Cell V(D)J Reagent Kits User Guide and loaded on the Chromium 10x platform using the 5′ v1.1 chemistry (10x Genomics). Libraries were sequenced on a NovaSeq4000 (Illumina, San Diego, CA, USA) to an average of ~50,000 reads per cell. Read alignment was carried out using the Cell Ranger pipeline against the mouse genome reference (mm10). Resultant cell by gene matrices for each sample were merged across all tested conditions and samples. Pre-processing, alignment and data filtering were applied equivalently to all samples using OpenPipeline v0.7.0 workflows (github.com/openpipelines-bio). Cells with less than 1000 UMIs, less than 200 genes or more than 25% mitochondrial counts were removed from downstream analysis. The downstream analysis was carried out in R using the Seurat v4.1.1 package [21].

Data were log-normalized with a scaling factor of 10,000. For the first level clustering, the top 2000 most variable genes were selected (‘vst’ method implemented in FindVariableFeatures()) and scaled using a linear model in the ScaleData() function. Afterwards, principal component analysis (PCA) was run, and the number of significant principal components (PCs) to be used for downstream cell clustering was determined using an ElbowPlot and heatmap inspection. The nearest neighbor graph and uniform manifold approximation and projection (UMAP) plot were generated using the significant PCs.

A Louvain clustering was run on all cells, and the best resolution for clustering was determined using an average silhouette scoring across all clusters, testing 10 resolutions between 0.1 and 1 as previously implemented in Ziegler et al. [22]. Marker genes for each cluster were calculated using the FindAllMarkers() function (method = ‘wilcox’) implemented in Seurat, and each cluster was iteratively subclustered further using the same approach. Sub-clustering was stopped when the resulting clusters were not meaningfully different. Clusters were annotated as cell-type populations based on the expression of genes that are known markers of specific cells by expert annotation and using the mouse liver atlas [23].

### 2.9. Single-Cell V(D)J Analysis

V(D)J (TCR and BCR) sequencing data were processed using the Cell Ranger V(D)J pipeline with mm10 as a reference. The downstream analysis was carried out in Python using the scirpy v0.12.2 library [24] and the dandelion v0.3.2 library [25], respectively, for TCR and BCR analysis. TCR and BCR annotations were further refined via V/D/J reannotation of constant region using IMGT’s mouse reference v3.1.38 [26], and only sequences corresponding to CH1 region for each constant gene/allele were retained. For the contigs assembled, low-confidence, non-productive or with UMIs < 2 were discarded. If five or more cells had identical dominant α–β pairs, along with no more than 15% mismatches in the CDR3 nucleotide sequences, the dominant α–β pairs were identified as clonal TCRs. Only cells that were annotated as T cells with the cell typing were considered for this analysis. Similarly, for B cells, a threshold of 3 cells was defined for clonality of BCRs. TCR and BCR sequencing data were processed using the Cell Ranger vdj pipeline with mm10 as a reference. For more in-depth BCR sequencing analysis, data obtained from the Cell Ranger pipeline were analyzed using the Immcantation toolkit [27,28]. Specifically, igblast V(D)J gene assignments were generated for each sample using the AssignGenes.py script, and a database for each sample was generated using MakeDb.py. Consequent to this, each sample was filtered to remove unproductive sequences and cells with multiple heavy chains using the Changeo R package, and cells were split by their heavy and light chains. For the samples, pairwise Hamming distances were estimated. We found that some samples had few clonal expansions, resulting in a small threshold distance cut-off. Consequently, we chose a manual threshold of 0.12 as the distance cut-off and combined the samples together for further clonal analysis. Hierarchical clustering was performed for the sequences that were within this distance threshold, and lineages were built using “buildPhylipLineage”. Finally, clonal trees and somatic hypermutation frequencies were inferred using the “observedMutations” command from the shazam R package, and trees were constructed using plotTrees in the alakazam R package. An R markdown script that contains the details of this analysis is available in the Supplementary Materials.

### 2.10. Statistical Analysis

Statistical comparisons were performed using either GraphPad Prism 9 or the package rstatix v0.7.2 in R. Statistical differences between the different treatment groups on immune responses and expression of HB antigen in liver were calculated using mixed-effect analysis and Tukey’s multiple comparison testing. Single-cell data differential proportion were calculated using a pairwise Wilcoxon test and FDR multiplicity adjustment (Benjamini–Hochberg) was carried out for all comparisons for each cell population. All *p*-values < 0.05 were considered significant. Due to small sample size, both raw and adjusted *p*-values are reported.

## 3. Results

### 3.1. Sequential Treatment with GalNAc-HBV siRNA and Therapeutic Vaccination Showed Sustained HBsAg Loss

To mirror the levels of HbsAg in CHB patients, we used the AAV-HBV mouse model with baseline HbsAg levels of 1000 IU/mL. Using this setting, we investigated whether lowering HbsAg and HbeAg levels for 6 weeks prior to vaccination (using a GalNAc-HBV siRNA) could improve vaccine efficacy levels. Four weeks after AAV-HBV transduction, once HbsAg levels were stable, mice were treated with three doses of GalNAc-HBV siRNA (n = 32) or control GalNAc-siRNA (n = 32) at 3-week intervals, followed by two or four doses (n = 16 active and 16 control siRNA per regimen) of pDNA vaccine at 3-week intervals starting on week 10 post transduction (Figure 1A).

Mice that received the control GalNAc-siRNA and the mock vaccine did not show any decline in viral parameters (Figure 1B,C and Figure S1). In contrast, GalNAc-HBV siRNA rapidly reduced serum HbsAg level by 2log_10_ IU/mL and HbeAg by >1 log_10_ IU/mL (Figure 1B,C, Figures S2 and S3). Animals that received GalNAc-control siRNA and then the therapeutic vaccine showed a moderate maximum decline of 1 log_10_ in HbsAg (Figure 1B,C and Figure S4).

**Figure 2:**
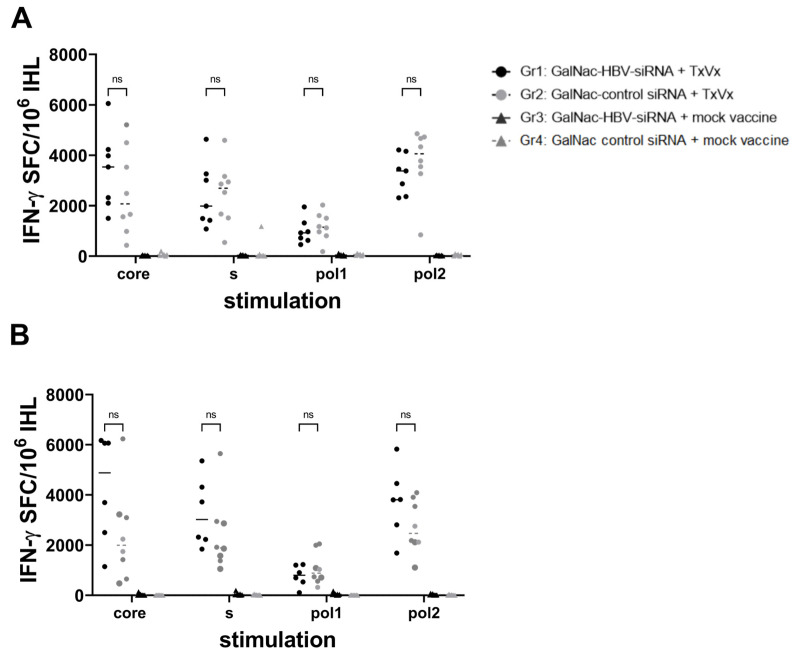
Induction of HBV-specific immune responses in AAV-HBV mice sequentially treated with GalNAc-siRNA and therapeutic vaccine. IHL from day 70 (1 week after second vaccination) (**A**) and from day 154 (7 weeks after last vaccination). (**B**) were stimulated directly ex vivo in IFN-g ELISpot plates with Core, Pol or surface peptide pools. Results are presented on the *y*-axis as spot-forming cells (SFC) per million of IHL, and the DMSO background is subtracted. Each dot or triangle is the mean of 3 replicates for each mouse. The horizontal line represents the median within one group. Black circles: GalNAc-HBV siRNA and TxVx; black triangles: GalNAc-HBV siRNA and mock vaccine; grey circle: GalNAc-control siRNA and TxVx; grey triangles: GalNAc-control siRNA and mock vaccine. *p* values are represented and calculated by Tukey’s multiple comparison test, ns means not significant.

GalNAc-HBV siRNA treatment alone failed to trigger HBV-specific immunity, measured by HbsAb titers (Figure 1D) and IFN-g ELISpot for Core, Pol or S (Figure 2), and consequently, viral relapse was observed 6 weeks after the last administration of siRNA. In contrast, all mice treated with GalNAc-HBV siRNA and therapeutic vaccine showed stable long-term (until day 154) loss of serum HbsAg, with the development of high titers of anti-HBs (Figure 1D) and induction of HBV-specific T cells (Figure 2), though the loss of HbeAg was only observed in some of the animals (Figure 1C and Figure S2D). Moreover, the reduction of HBV antigenemia through the sequential treatment with GalNAc-HBV siRNA prior to therapeutic vaccination was well tolerated with no effects on body weight and only a moderate and transient ALT elevation detected after the first vaccine (<100 mIU/mL, Figure 1E).

An important aspect of this study is the analysis of the liver immune environment in the context of different baseline levels of HBV and upon (or no) treatment. In concordance with the HBsAg serum levels data, the levels of HBsAg in the liver 1 week after the second vaccination (day 70) were reduced upon treatment with GalNAc-HBV siRNA, irrespective of whether they were subsequently vaccinated or not (*p* = 0.0096, *p* = 0.0162, respectively, Figure 1F). As described before, no reduction in HBsAg levels was observed in those mouse groups treated with GalNAc-control siRNA, either vaccinated from week 6 or not.

At day 154 (7 weeks after the last vaccination), the HBsAg levels in the liver remained low for the groups treated with GalNAc-HBV siRNA, irrespective of their vaccination status. Moreover, multiple vaccinations also had an effect on HBsAg in the liver since mice treated with GalNAc-control siRNA and therapeutic vaccine showed a further decline in HBsAg. For HBcAg in the liver, a comparable profile was seen as described for HBsAg (Figure 1G). At day 70, treatment with GalNAc-HBV siRNA had a significantly decreasing effect on the expression of HBcAg in the liver, irrespective of vaccination. At day 154, the HBcAg levels remained low in the groups treated with GalNAc-HBV siRNA, whereas those treated with control siRNA and therapeutic vaccine showed a decrease in HBcAg (Figure 1G). These data corroborate the previous data that suggest that (higher) efficacy of therapeutic vaccination requires levels of HBsAg <100 IU/mL. We showed that an HBV siRNA is an effective agent in reducing HBsAg levels and enables and improves the efficacy of the therapeutic vaccine.

To investigate and identify potential correlates of protection, the induction of liver HBV-specific T cells was evaluated 1 week after the second vaccination (day 70, Figure 2A) and 7 weeks after the last vaccination (day 154, Figure 2B). HBV-specific T cells, measured by IFN-g ELISPOT, could be detected in the livers of all mouse groups vaccinated with HBV-expressing pDNA. At day 154 after the start of treatment, the strength of HBV-specific immune responses increased against all HBV antigens compared to responses at day 70. Taken together, these data show that treatment with GalNAc-siRNA prior to therapeutic vaccination enables a high level of HBV-specific T-cell responses. Mice vaccinated with mock vaccine did not show any induction of HBV-specific T cells irrespective of their treatment with GalNAc-siRNA.

### 3.2. Liver CD4 Tfh-like Cell Subpopulation Induced by Vaccination, as Identified by Single-Cell RNA Sequencing Analysis

**Figure 3:**
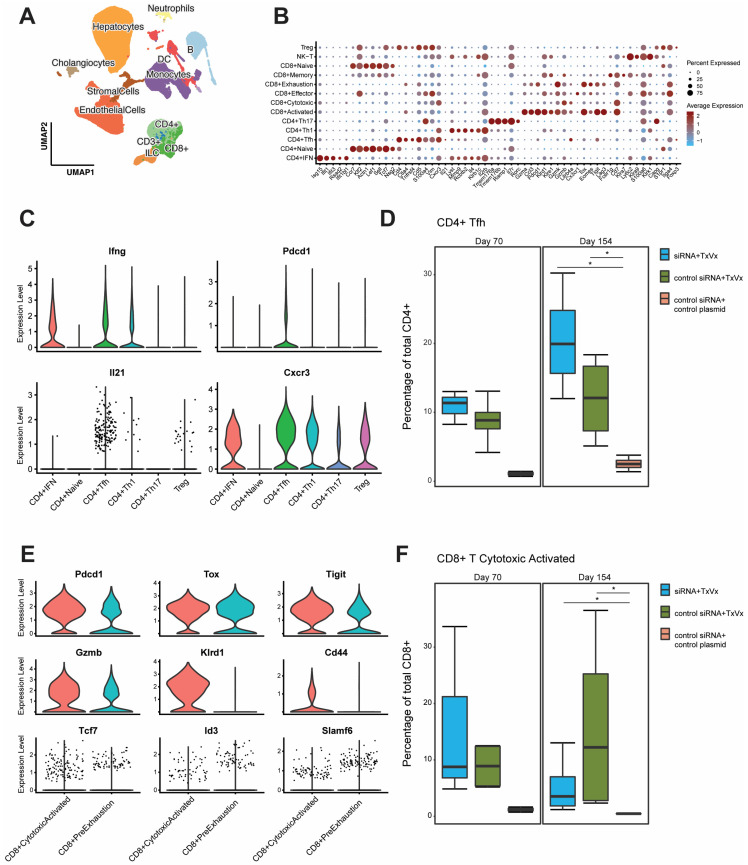
Single-cell liver atlas of the AAV-HBV model. (**A**) Overview of the full liver single-cell RNA-seq atlas using UMAP highlighting the top-level cell annotation. (**B**) Dot plot of top differentially expressed genes across the T-cell compartment. (**C**) Violin plot from CD4 Tfh genes across the CD4 T-cell compartment. (**D**) Box plot of CD4 Tfh-cell percentages in total CD4 T cells in the different groups faceted across the two sampling timepoints. (**E**) Violin plot from effector and exhaustion genes in CD8 cytotoxic activated and CD8 pre-exhaustion. (**F**) Box plot of CD8 cytotoxic activated cell percentages in total CD8 T cells in the different groups faceted across the two sampling timepoints. Pairwise Wilcoxon test, with fdr correction; comparison testing (* *p* < 0.05).

To identify which liver cells are most relevant to break T-cell tolerance, single-cell RNA sequencing was performed to generate a liver cells atlas. Liver resident cells from three groups of mice transduced with 2.5 × 10^9^ vge of AAV-HBV and treated with either GalNAc-HBV siRNA and therapeutic vaccine, GalNAc-control siRNA and therapeutic vaccine or control GalNAc-siRNA and mock vaccine (mock group) were analyzed using single-cell RNA sequencing 1 week after the second vaccine administration (day 70, first timepoint, Figure 1A) and 7 weeks after the fourth vaccine administration (day 154, second timepoint, Figure 1A). A total of four samples per group per timepoint were profiled, except for the first timepoint of the GalNAc-HBV siRNA and therapeutic vaccine and the control groups (Figure S5A). The uneven sample size between the first and second timepoints hampered a meaningful statistical comparison. After filtering for quality and doublet identification during cell typing, 250,966 cells across a total of 22 samples were obtained. Across all mouse liver samples, the lowest number of cells obtained was 8590, and the maximum was 15,623 (Figure S5A). After annotation, 11 major cell types were obtained: B cells, T cells, cholangiocytes, dendritic cells, endothelial cells, granulocytes, hepatocytes, innate lymphoid cells (ILCs), monocytes, neutrophils and stromal cells. The majority of the cells detected across the full dataset were liver sinusoidal endothelial cells (LSECs) (27%) and hepatocytes (24%). B cells (8%), T cells (16%) and monocytes (14%) were also well represented, and the remaining cell types made up a smaller fraction of the data (Figure 3A; Table S1). The major subpopulations were further annotated to provide more granularity to each cell type (Figure S5B), leading to a total of 39 subpopulations. Within the T-cell compartment, a total of 13 subpopulations (Figure 3B) were identified, representing CD8, CD4 and NK-T cells. Of note, within the CD4 compartment, a subpopulation with the phenotype of a T-follicular helper-like cell expressing *Pdcd1* (PD-1), *Ifng*, *Cxcr3*, *Il21* and *Cd40lg* (Figure 3C) was identified. This subpopulation was significantly increased (raw *p*-value = 0.029, adjusted *p*-value = 0.044) in both vaccinated groups versus mock vaccinated group on day 154, though an increased trend at day 70 was already observed (Figure 3D).

### 3.3. Decrease in Liver Naïve CD8 T Cells and Increase in Pre-Exhausted Naïve Cells upon Vaccination

The CD8 compartment comprised of naïve, central memory, effector memory, cytotoxic T cells and two populations expressing *Pdcd1* (PD-1), *Tox*, *Gzmk* and *Tigit*: cytotoxic activated and pre-exhausted T cells (Figure 3B,E). The cytotoxic activated cells were distinguished from the pre-exhausted population based on their cytotoxic phenotype and their expression of *Cd44*, *Klrd1* and higher levels of *Gzmb* (Figure 3E). This cytotoxic activated CD8 population and CD8 pre-exhausted T cells were significantly increased upon vaccination irrespective of treatment with siRNA at day 154 versus the mock group (raw *p*-value = 0.026, adjusted *p*-value = 0.039, Figure 3F; Figure S6A, raw *p*-value = 0.029, adjusted *p*-value= n.s., respectively). In contrast, the central memory T-cell compartment showed a significantly decreased frequency in the vaccine-treated groups, irrespective of treatment with siRNA, compared to the mock-treated group at day 154 (Figure S6A; raw *p*-value = 0.029, adjusted *p*-value = 0.044). Frequency comparison between the treatment groups showed a trend (albeit non-significant) of decreased naïve T cells in both vaccinated groups irrespective of siRNA in comparison with the mock group (Figure S6A). Finally, the cytotoxic T cells showed increased frequency only in the group that received GalNAc-HBV siRNA and subsequent HBV DNA vaccination, but not in the GalNAc-control siRNA and therapeutic vaccine group compared to the mock-treated group at day 154 (Figure S6A, raw *p*-value = 0.029, adjusted *p*-value= n.s.).

To assess their proliferation capacity, we looked at *Tcf7* (TCF-1) and *Id3* expression [29,30]. Both populations (pre-exhausted and cytotoxic activated) expressed *Tcf7* in the treatment groups, albeit at low levels (Figure 3E). In the pre-exhausted T cells, the expression of *Tcf7* and *Id3* was higher in the vaccine groups compared to the mock vaccinated group (Figure S6B).

### 3.4. Macrophage, NK Cell and Neutrophil Compartments Change upon Therapeutic Vaccination

**Figure 4:**
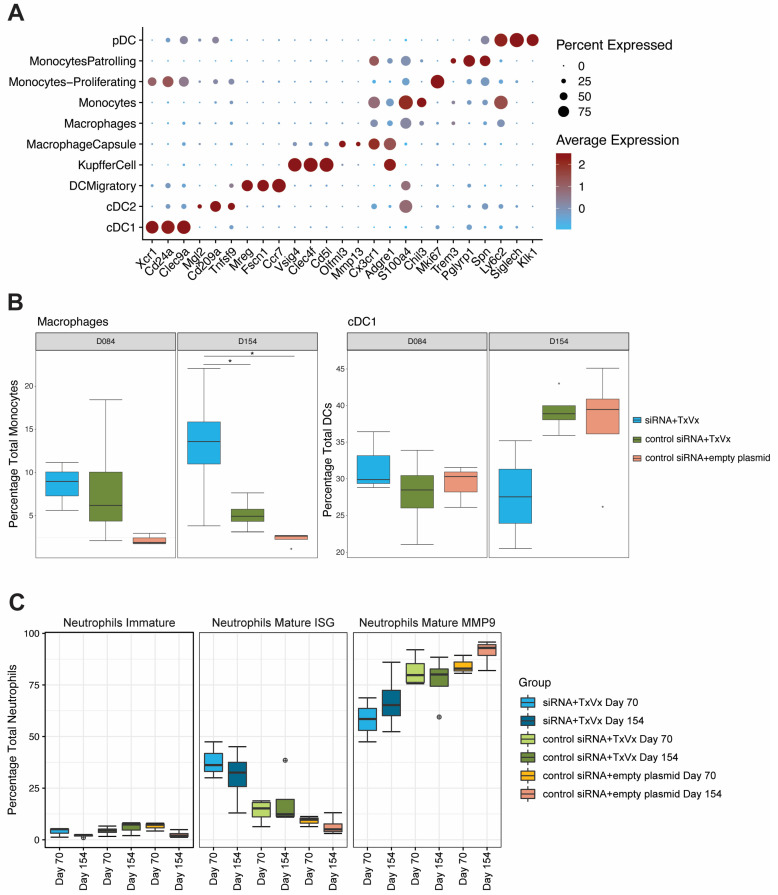
**Monocyte, DC and neutrophil compartment frequency analysis.** AAV-HBV-transduced C57BL/6 mice were treated with GalNAc-HBV siRNA and therapeutic vaccine, with GalNAc-control siRNA and therapeutic vaccine, and with GalNAc-control siRNA and mock vaccine. Intrahepatic immune cells from the liver were isolated 1 week after the administration of the second therapeutic vaccine (timepoint day 70) or 7 weeks after the fourth therapeutic vaccine (timepoint day 154). (**A**) Dotplot from scaled expressed hallmark genes for the antigen-presenting cell and monocyte compartments. (**B**) Frequency of macrophages across groups and statistical comparison at day 154 (paired Wilcox test, non-parametric, fdr adjustment). Macrophages at day 154 from GalNAc-HBV siRNA and therapeutic vaccine vs. mock were significant (*p* = 0.029, *p*.adj = 0.0435), and from GalNAc-HBV siRNA and therapeutic vaccine vs. GalNAc-control siRNA and therapeutic vaccine (*p* = 0.029, *p*.adj = 0.0435). Frequency of cDC1 across groups and statistical comparison at day 154 (paired Wilcox test, non-parametric, fdr adjustment). cDC1 at day 154 from GalNAc-HBV siRNA and therapeutic vaccine vs. GalNAc-control siRNA and therapeutic vaccine were significant (*p* = 0.029, *p*.adj = 0.087). (**C**) Frequency of neutrophils across groups and statistical comparison at day 154 (paired Wilcox test, non-parametric) with fdr adjustment showed no significant comparisons. Significant comparisons are highlighted with *. Boxplot outlier values are represented by a circle.

Within the monocyte and dendritic cell compartments, we identified four subtypes of dendritic cells (cDC1, cDC2, migratory DCs and pDCs, as described in [23]), three subtypes of macrophages (Kupffer cells, capsule macrophages and macrophages) and three subtypes of monocytes (classical, patrolling and proliferating monocytes), which were annotated according to their hallmark genes in Figure 4A. A higher frequency of macrophages was observed within the two vaccinated groups irrespective of siRNA treatment versus the mock group (Figure 4B; raw *p*-value = 0.029, adjusted *p*-value = 0.044) at the second timepoint sampled (day 154). No changes were observed in Kupffer cells (Figure S7A).

As previously mentioned, even though the uneven sample size between the first and second timepoints hampered a statistical comparison, we observed that the frequency of cDC1s remained stable across the two timepoints when GalNAc-HBV siRNA treatment was followed by therapeutic vaccination. In contrast, both the mock group and the group that received control-siRNA and therapeutic vaccine showed a trend of increased frequency at day 154 compared to day 70 (Figure 4B, raw *p*-value = 0.029, adjusted *p*-value = 0.087). The cDC2 population showed an increased trend at day 154 only in the GalNAc-HBV siRNA, followed by the therapeutic vaccine group, whereas it remained comparable in the other groups. Across the three treatment arms, a decrease in frequency on day 154 was observed in the pDCs (Figure S7B) compared to day 70. Migratory DCs at day 154 were significantly increased in GalNAc-HBV siRNA, followed by the vaccine group versus the mock vaccine group (Figure S7B, raw *p*-value = 0.029, adjusted *p*-value = 0.057).

The neutrophil compartment, despite having low numbers of cells, showed interesting trends. We were able to detect three populations: an immature population expressing *Ltf*, *Lcn2* and *Camp* and two mature populations (Figure S8A): one of them expressing typical type I IFN signature genes such as *Isg15*, *Mme*, *Rsad2* and *Cd274* (PD-L1); the other expressing immunosuppressive markers like *Mmp8*, *Mmp9*, *Arg1* and *Padi4* (Figure S8B). In addition, the mature populations also showed an interesting trend across the different arms, where the GalNAc-HBV siRNA followed by the therapeutic vaccination group showed higher frequency of the type I IFN (ISG) neutrophils and lower immunosuppressive (MMP9) neutrophils than both the control siRNA and therapeutic vaccine groups and the mock group (Figure 4C).

In the NK compartment, the population CD11b+CD27- (high cytolytic function) was increased at day 154 in the groups not receiving GalNAc-HBV siRNA (Figure S9A; raw *p*-value = 0.029, adjusted *p*-value = 0.087). This showed an inverted trend with the CD11b-CD27+ cells with a cytokine-producing phenotype. CD11b+CD27- cells expressed high levels of *Gzmb*, *Prf1*, *Nkg7*, whereas CD11b-CD27+ expressed *Cd160* and *Il2* (Figure S9B).

### 3.5. Therapeutic Vaccination Induces TCR Clonal Expansion across Multiple T-Cell Subtypes

To investigate whether our vaccination strategy induced clonality in the T-cell compartment, single-cell TCR sequencing was performed. The data-driven cell type annotation was paired with the TCR repertoire clonality for further granularity (Figure S10A,B). The CD8 T-cell compartment showed clonal TCR expansion in both groups receiving vaccination irrespective of siRNA treatment. The pre-treatment with GalNAc-HBV siRNA and subsequent therapeutic vaccination group showed 33% of the CD8 T cells with clonal expansion at the first timepoint (day 70), increasing to 53% at the second timepoint (day 154). The group treated with GalNAc-control siRNA followed by therapeutic vaccination showed 31% of the CD8 T cells with clonal expansion at the first timepoint (day 70) and 39% at the second timepoint (day 154). For the mock group, it was observed at 1 and 2%, respectively (Table S2).

**Figure 5:**
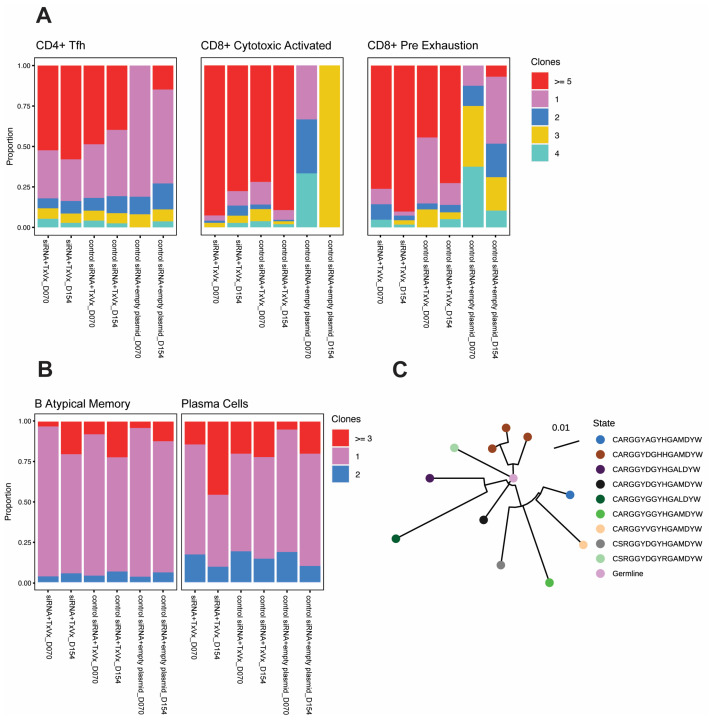
**TCR and BCR clonality.** (**A**) Proportion of CD4 Tfh, CD8 cytotoxic activated and CD8 pre-exhaustion with clonal expansion (represented by ≥5 clones). (**B**) Proportion of B atypical and plasma cells with clonal expansion (represented by ≥3 clones). (**C**) Clonal phylogenetic analysis from CDR3 sequences from a siRNA and therapeutic vaccine sample.

When looking into each CD8 T-cell subtype, CD8 naïve and CD8 central memory T cells did not show expansion across the different groups (Figure S10B). Interestingly, the CD8 T-cell population with the cytotoxic activated phenotype (expressing *Pdcd1*, *Gzmb*, *Klrd1* and *Cd44*) showed over 70% clonality in the groups that received the therapeutic vaccine irrespective of siRNA treatment and independent of the number of vaccines received (Figure 5A). The pre-exhaustion population showed high clonality in the group that received GalNAc-HBV siRNA followed by therapeutic vaccine, with 76% of cells clonally expanded in the first timepoint (day 70) and 90% in the second timepoint (day 154). For the group treated by GalNAc-control siRNA followed by therapeutic vaccine, the first timepoint showed 44% clonal expansion and increased to 73% at the second one (Figure 5A).

In the CD4 T-cell compartment, a more modest expansion was observed and mainly driven by the CD4 Tfh cells (Figure 5A and Figure S10A). On a global CD4 T-cell level, the GalNAc-HBV siRNA, followed by the therapeutic vaccine group, showed 8% clonal expansion at the first timepoint (day 70) and 23% at the second timepoint (day 154). The GalNAc-control siRNA group, followed by the therapeutic vaccine group, showed 9% at both timepoints. For the mock group, it showed 3 and 2%, respectively (Table S3).

When breaking down the clonal expansion based on the transcriptional profile cell annotation, for the CD4 Tfh, the group treated with GalNAc-HBV siRNA and subsequent therapeutic vaccine remained stable with 52 and 58% clonal expansion at both timepoints. For the group treated with GalNAc-control siRNA followed by therapeutic vaccination, clonality reached 49 and 40% at both timepoints, respectively. In the mock group, no clonal expansion was detected at the first timepoint, while 15% was detected at the second (Figure S10A). Interestingly, the Treg compartment showed no clonal expansion in the mock group, while 14% at the first timepoint and 12% clonality at the second timepoint were observed for the group that received GalNAc-HBV siRNA followed by therapeutic vaccination. The group receiving GalNAc-control siRNA and therapeutic vaccine showed 5 and 7% clonality, respectively (Figure S10A).

### 3.6. BCR Clonal Expansion across Atypical B Cells and Plasma Cells

Four different B-cell subpopulations were detected: activated B cells expressing *Myc*, *Nr4a1*, *Ier2* and *Nfkbid*; classical memory B cells expressing *Ighd* and *Fcer2a*; atypical memory B cells expressing *Fcrl5* and *Lgals1*; and finally plasma cells expressing *Jchain*, *Xbp1* and *Igha* (Figure S11A). We observed BCR clonal expansion only in atypical memory B and plasma cells but not in activated B cells and classical memory B cells (Figure 5B). Plasma cells sampled from mice receiving GalNAc-HBV siRNA followed by therapeutic vaccine showed a proportion increase in clonal expanded cells from 14% at the first timepoint (day 70) to 46% at day 154. The group receiving control siRNA followed by therapeutic vaccine showed 20% and 22% clonality at each timepoint, respectively. Within the atypical B-cell subset, both treatment groups reached 20% clonality by the second timepoint (Figure S11B).

To understand whether the clonally expanded plasma cells expressed long-lived plasma cell genes [31], we checked for *Sdc1* (encoding CD138), *Tnfrsf17* (encoding BCMA) and *Epcam* (encoding CD326). A lower expression of all genes was observed in plasma cells with only one clone (Figure S11C). Correspondingly, no clonal expansions were detected in the absence of vaccination. All phylogenetic analyses are described in Figure 5C and Figure S11D–F.

## 4. Discussion

Despite considerable scientific advancements in the field of HBV therapeutic vaccination, the key factors essential for breaking HBV tolerance continue to be elusive. As of today, no well-established immunological markers are available that would help guide treatment and monitor cases for stopping antiviral therapy. The current study provides novel insights into the immunological effect that lowering HBV levels has on the liver immune environment, using the AAV-HBV mouse model in a setting mirroring the HBsAg level observed in CHB patients. Our approach provides novel insights (at a single cell level) into the dynamics in the liver microenvironment upon vaccination and the effect of lowering HBsAg levels through treatment with GalNAc-HBV siRNA prior to therapeutic vaccination. This provides insightful information for potential therapeutic approaches.

Using a pDNA-based vaccine encoding for Core, Pol and S antigens, we demonstrated that high-level HBsAg expression correlated with HBV-specific immune tolerance in AAV-HBV mice, preventing successful therapeutic vaccine efficacy and HBsAg clearance, as previously described [14]. The inhibition of HBV antigen expression in hepatocytes using a liver-directed GalNAc-HBV siRNA enabled a therapeutic vaccine approach to elicit its full immunogenic capacity and led to sustained HBsAg loss in mice with HBsAg levels around 3log_10_ IU/mL or less [14]. This was accompanied by the induction of both HBV-specific T- and B-cell responses, likely driving the sustained suppression of HBsAg. The induction of both HBV-specific CD4 and CD8 T cells accompanied by neutralizing antibodies is also seen in the human setting in resolver patients [8].

The observed enhancement of HBsAb in AAV-HBV mice could be a concern since the induction of anti-HBs could act as a decoy for measuring HBsAg in serum, which could bias its detection. To circumvent this, in this study, HBsAg levels were measured in both serum and liver. Concomitant with the decline in the serum, HBsAg was also decreased in the liver of animals that received the vaccine alone, clearly indicating a treatment effect. Furthermore, in the group that received the GalNAc-HBV siRNA followed by therapeutic vaccination, a further reduction of HB Core antigen in the liver was observed, which was accompanied by a transient and small ALT increase after the first vaccination. Taken together, these findings clearly indicate that there is a vaccine effect on HBV antigens, likely by induction of T cells driven by the HBsAg levels in serum and liver.

To further understand the immune microenvironment in the liver during vaccination and whether prior lowering of HBsAg GalNAc impacted liver immune dynamics, single-cell RNA and V(D)J sequencing were performed. Though limited by a small sample size, the high number of cells per sample allowed for an in-depth cell population analysis. Mice treated sequentially with GalNAc-HBV siRNA and therapeutic vaccine cleared HBV antigenemia, which correlated both with strong CD4 Tfh responses and TCR clonal expansions, as well as plasma cell clonal expansion. In contrast, mice treated with control siRNA (that therefore maintained high levels of HBV antigenemia) followed by therapeutic vaccination did not all clear HBV antigenemia despite strong induction of CD4 Tfh and TCR clonal expansions. Given that the main difference with the group that cleared HBV was a more modest plasma cell clonal expansion, it suggests that mounting a CD4 Tfh response alone is not sufficient for clearing HBV and that induction of B cells is a key factor [32].

It is well established that CD4 Tfh cells are key in supporting germinal center B cells to produce vaccine-specific immunoglobulins [33], and CD4 T-cell priming has been reported as critical for a successful therapeutic vaccination in AAV-HBV mice with a recombinant protein prime/MVA boost [34]. Our study shows that anti-HBs were detectable in serum, starting at 70 days post treatment. CD4 Tfh cells were detected already on the first single-cell analysis timepoint (day 70) in both vaccinated groups, indicating that these were able to support B cells mounting a productive antibody response. The B-cell compartment showed the highest clonal expansion at day 154, especially within plasma cells of mice treated with both GalNAc-HBV siRNA and therapeutic vaccine. Correspondingly, upon phylogenetic reconstruction, no clonally expanded lineages were detected for B cells for any of the treated groups at the early timepoint (day 70). For day 154, we found clonally expanded lineages, with greater clonal expansion in both groups that received the vaccine.

In the CD8 T-cell compartment, the cytotoxic activated population, expressing PD-1, as well as effector markers, was significantly increased in both vaccinated groups in comparison with the control group. Moreover, these showed pronounced clonal expansion, indicating a vaccination-related activation. Although the differences between mice that received the vaccine alone and those that received the sequential treatment of GalNAc-HBV siRNA and therapeutic vaccine were not significant, there was a clear trend of more TCR clonal expansion in the dual treatment group over time compared to the vaccine-only group. Combining these data with the ELISpot data leads us to suggest that the induction of both T and B cells is important, as is the function and clonal expansion of these cells, which represents a correlate of protection.

Besides effector markers, *Pdcd1* (*PD-1*) and *Tox* were also upregulated on CD8 T cells, which warrants further monitoring to understand whether these cells might progress to a terminal exhaustion phenotype or whether this is a transient effect of T-cell activation. The cells expressing *Pdcd1* and *Tox* also expressed *Tcf7*, *Id3* and *Slamf6*; therefore, they were not terminally exhausted and indicated a potential transient state. A previous report from our group [19] has shown terminal exhaustion in AAV-HBV mice with highly stable HBsAg levels (4log_10_IU/mL); therefore, we postulate that the mice with lower stable HBsAg levels (2–3log_10_IU/mL) used in this study did not elicit a terminal exhausted phenotype [19]. Nonetheless, the potential benefit of treatment with immune checkpoint inhibitors is being explored [35].

Lastly, in the neutrophil compartment, some interesting trends were observed. The mature neutrophil population MMP9, expressing immunosuppressive markers, showed higher frequency in the control group and therapeutic vaccine alone group compared to the mice that received the sequential treatment. These neutrophils have been stated to have potent immunosuppressive capabilities in oncology [36]. The same might be true in the context of chronic infection, where it has been described that HBV may reduce neutrophil responses [37]. The more activated neutrophils (expressing *Isg15*) showed the opposite trend, with higher frequencies in the treated groups, which could indicate that these might be mediating infected hepatocyte killing. A similar neutrophil cell type expressing type I interferon activation has already been described in chronic hepatitis B patients [16], pointing towards possible translation in humans.

## 5. Conclusions

This study provides novel insight into the immune changes in the liver at the single-cell level, showing a correlation between the treatment-induced reduction in HBsAg levels and the clonal expansion of CD4 follicular helper T cells, CD8 cytotoxic T cells, plasma cells and ISG-producing neutrophils in the liver upon HBV siRNA and subsequent therapeutic vaccine treatment. More studies are required to identify new immune approaches in HBV treatment that could lead to a functional cure after low HBsAg levels are achieved.

## Data Availability

Single-cell RNA-seq data (raw and processed counts) are available in Zenodo with the following accession number: 10217383.

## Supplementary Materials

The following supporting information can be downloaded at https://www.mdpi.com/article/10.3390/vaccines11121825/s1. Figure S1: Viral parameters over time of individual mice that were initially transduced with 2.5 × 109 vg/mL rAAV-HBV and treated with GalNAc-control siRNA and mock vaccine. Figure S2: Viral parameters over time of individual mice that were initially transduced with AAV-HBV (2.5 × 109 vg/mL) and treated with GalNAc-HBV siRNA and therapeutic vaccine (TxVx). Figure S3: Viral parameters over time of individual mice that were initially transduced with 2.5 × 109 vg/mL rAAV-HBV and treated with GalNac-HBV siRNA and mock vaccine. Figure S4: Viral parameters over time of individual mice that were initially transduced with 2.5 × 109 vg/mL rAAV-HBV and treated with GalNAc-control siRNA and therapeutic vaccine (TxVx). Figure S5: Single-cell RNA sequencing QC and annotation. Figure S6: CD8 T-cell frequency and pre-exhaustion profiles. Figure S7: Monocyte and DC compartment frequency analysis. Figure S8: ILC compartment frequency analysis. Figure S9: Neutrophil compartment analysis. Figure S10: TCR clonality frequency. Figure S11: B-cell compartment analysis. Table S1: Percentage distribution of cell types. Table S2: Percentage distribution of CD8 T-cell clonal expansion. Table S3: Percentage distribution of CD4 T-cell clonal expansion.
