# Supplementary material for: Sustained Liver HBsAg Loss and Clonal T- and B-Cell Expansion upon Therapeutic DNA Vaccination Require Low HBsAg Levels
Source: Vaccines (Basel). 2023 Dec 6;11(12):1825. doi: 10.3390/vaccines11121825 (PMC10747285; doi:10.3390/vaccines11121825)
Supplement: Supplementary file 1 [file vaccines-11-01825-s001.zip › vaccines-2716206-supplementary.pdf]

SUPLEMMENTARY Appendix:

Table of contents

Supplemental results:

Figure S1. Viral parameters over time of individual mice that were initially transduced with 2.5x10^9vg/ml rAAV-HBV and treated with GalNAc-control siRNA and mock vaccine.

Figure S2. Viral parameters over time of individual mice that were initially transduced with AAV-HBV (2.5x10^9vg/ml) and treated with GalNAc-HBV siRNA and therapeutic vaccine (TxVx).

Figure S3. Viral parameters over time of individual mice that were initially transduced with 2.5x10^9vg/ml rAAV-HBV and treated with GalNac-HBV siRNA and mock vaccine.

Figure S4. Viral parameters over time of individual mice that were initially transduced with 2.5x10^9vg/ml rAAV-HBV and treated with GalNAc-control siRNA and therapeutic vaccine (TxVx).

Figure S5. Single-cell RNA sequencing QC and annotation.

Figure S6. CD8 T cell frequency and pre-exhaustion profiles.

Figure S7. Monocyte and DC compartment frequency analysis.

Figure S8. ILC compartment frequency analysis.

Figure S9. Neutrophil compartment analysis.

Figure S10. TCR clonality frequency.

Figure S11. B cell compartment analysis.

Supplementary Table S1 – Percentage distribution of cell types

Supplementary Table S2 – Percentage distribution of CD8 T cell clonal expansion

Supplementary Table S3 – Percentage distribution of CD4 T cell clonal expansion

Figure S1- Viral parameters over time of individual mice that were initially transduced with $2.5x10^{9}$vg/ml rAAV-HBV and treated with GalNAc-control siRNA and mock vaccine. Individual-level data are shown; the data corresponds to the mean data plotted in Figure 1. **(A)** Hepatitis B surface antigen levels (HBsAg levels) in IU/ml measured in serum until day 70. **(B)** HBsAg levels in IU/ml measured in serum until day 154. **(C)** Hepatitis B e antigen levels (HBeAg) in IU/ml measured in serum until day 70. **(D)** HBeAg levels in IU/ml measured in serum until day 154. **(E)** Hepatitis B surface antibody levels (HBsAb) in mIU/ml measured in serum until day 70 **(F)** HBsAb levels in mIU/ml measured in serum until day 154. **(G)** alanine aminotransferase (ALT) activity in mU/ml measured in serum until day 70. **(H)** ALT levels measured in serum until day 154.

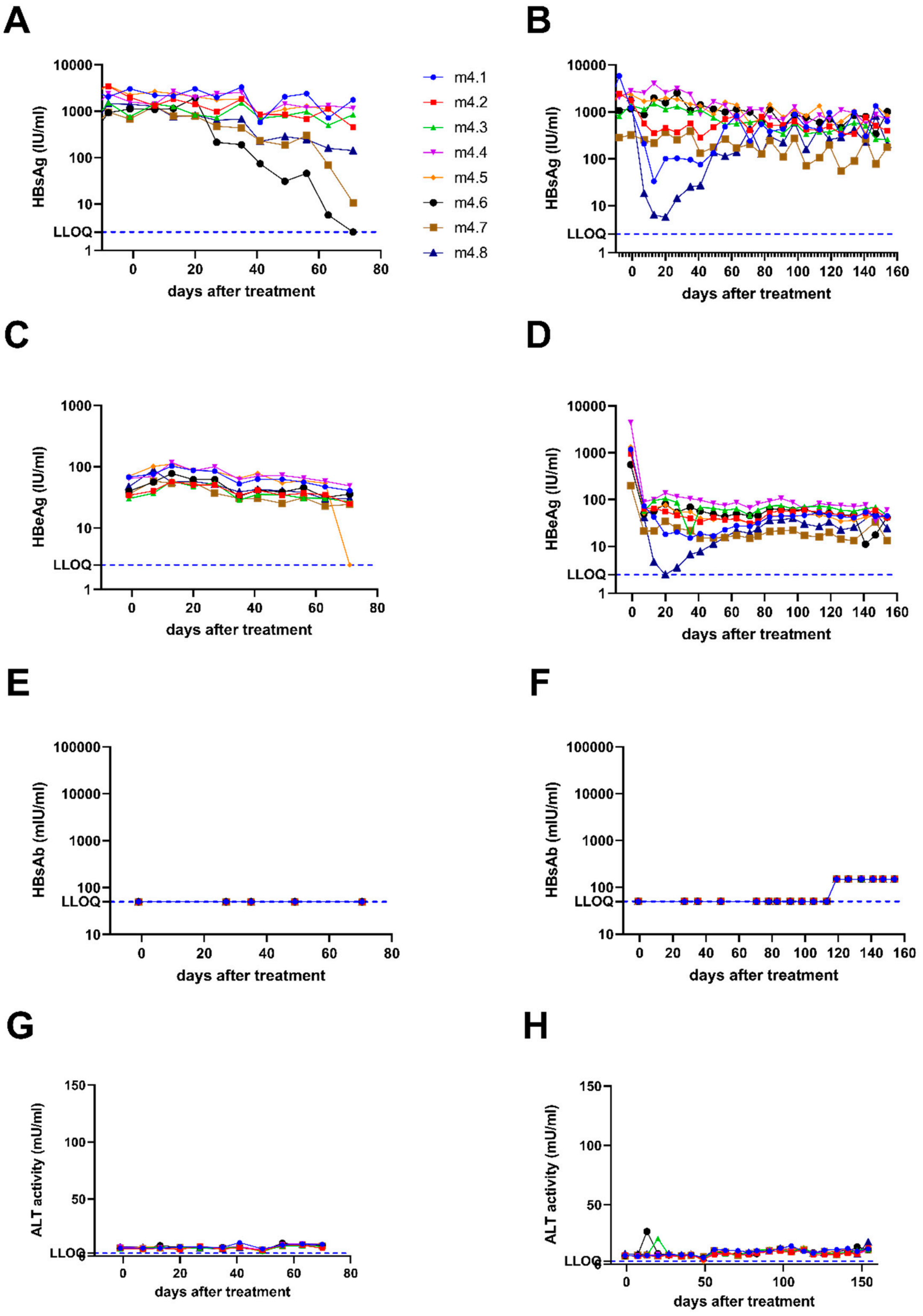

Figure S2- Viral parameters over time of individual mice that were initially transduced with AAV-HBV (2.5x10^{9}vg/ml) and treated with GalNAc-HBV siRNA and therapeutic vaccine (TxVx). Individual-level data are shown; the data corresponds to the mean data plotted in Figure 1. **(A)** Hepatitis B surface antigen levels (HBsAg levels) in IU/ml measured in serum until day 70. **(B)** HBsAg levels in IU/ml measured in serum until day 154. **(C)** Hepatitis B e antigen levels (HBeAg) in IU/ml measured in serum until day 70. **(D)** HBeAg levels in IU/ml measured in serum until day 154. **(E)** Hepatitis B surface antibody levels (HBsAB) in mIU/ml measured in serum until day 70 (F) HBsAb levels in mIU/ml measured in serum until day 154**. (G)** alanine aminotransferase (ALT) activity in mU/ml measured in serum until day 70. **(H)** ALT levels measured in serum until day 154. Dotted blue line represent the lower limit of quantification (LLOQ) of the assay.

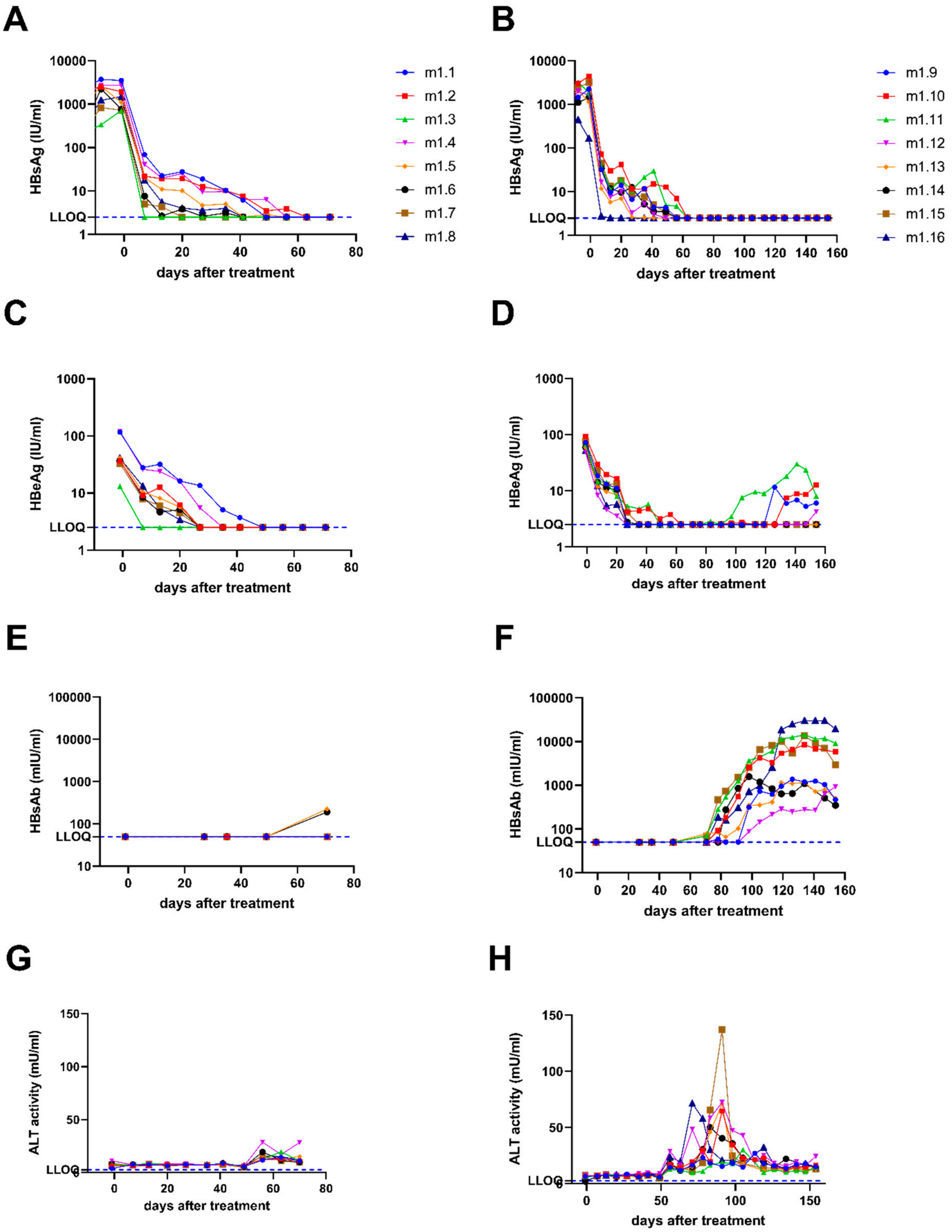

Figure S3- Viral parameters over time of individual mice that were initially transduced with 2.5x10^{9}vg/ml rAAV-HBV and treated with GalNAc-HBV siRNA and mock vaccine. Individual-level data are shown; the data corresponds to the mean data plotted in Figure 1. **(A)** Hepatitis B surface antigen levels (HBsAg levels) in IU/ml measured in serum until day 70. **(B)** HBsAg levels in IU/ml measured in serum until day 154. **(C)** Hepatitis B e antigen levels (HBeAg) in IU/ml measured in serum until day 70. **(D)** HBeAg levels in IU/ml measured in serum until day 154. **(E)** Hepatitis B surface antibody levels (HBsAb in mIU/ml measured in serum until day 70 **(F)** HBsAb levels in mIU/ml measured in serum until day 154. **(G)** alanine aminotransferase (ALT) activity in mU/ml measured in serum until day 70. **(H)** ALT levels measured in serum until day 154.

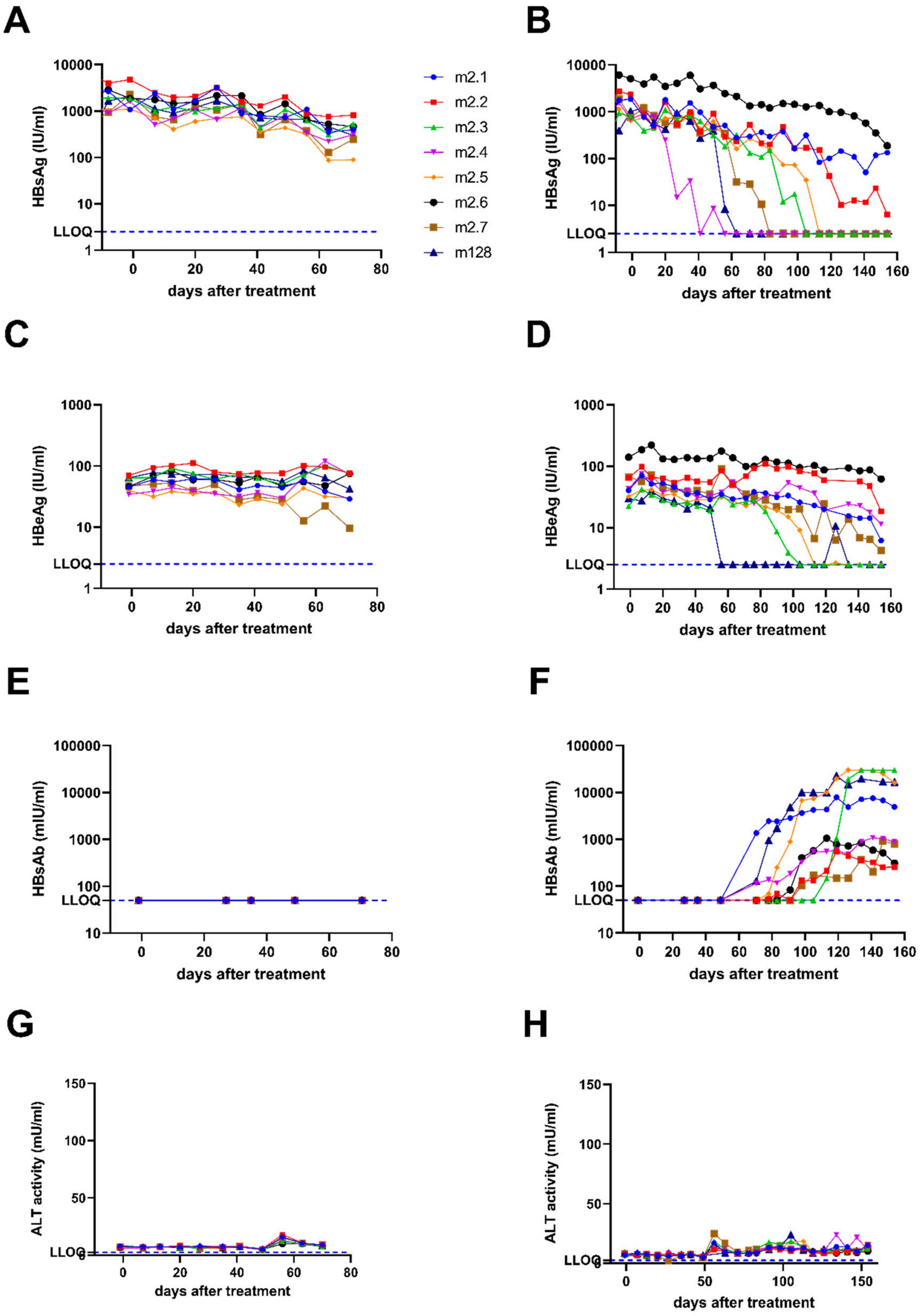

Figure S4- Viral parameters over time of individual mice that were initially transduced with 2.5x10^{9}vg/ml rAAV-HBV and treated with GalNAc-control siRNA and therapeutic vaccine. Individual-level data are shown; the data corresponds to the mean data plotted in Figure 1. **(A)** Hepatitis B surface antigen levels (HBsAg levels) in IU/ml measured in serum until day 70. **(B)** HBsAg levels in IU/ml measured in serum until day 154. **(C)** Hepatitis B e antigen levels (HBeAg) in IU/ml measured in serum until day 70. **(D)** HBeAg levels in IU/ml measured in serum until day 154. **(E)** Hepatitis B surface antibody levels (HBsAB) in mIU/ml measured in serum until day 70 **(F)** HBsAb levels in mIU/ml measured in serum until day 154. **(G)** alanine aminotransferase (ALT) activity in mU/ml measured in serum until day 70. **(H)** ALT levels measured in serum until day 154

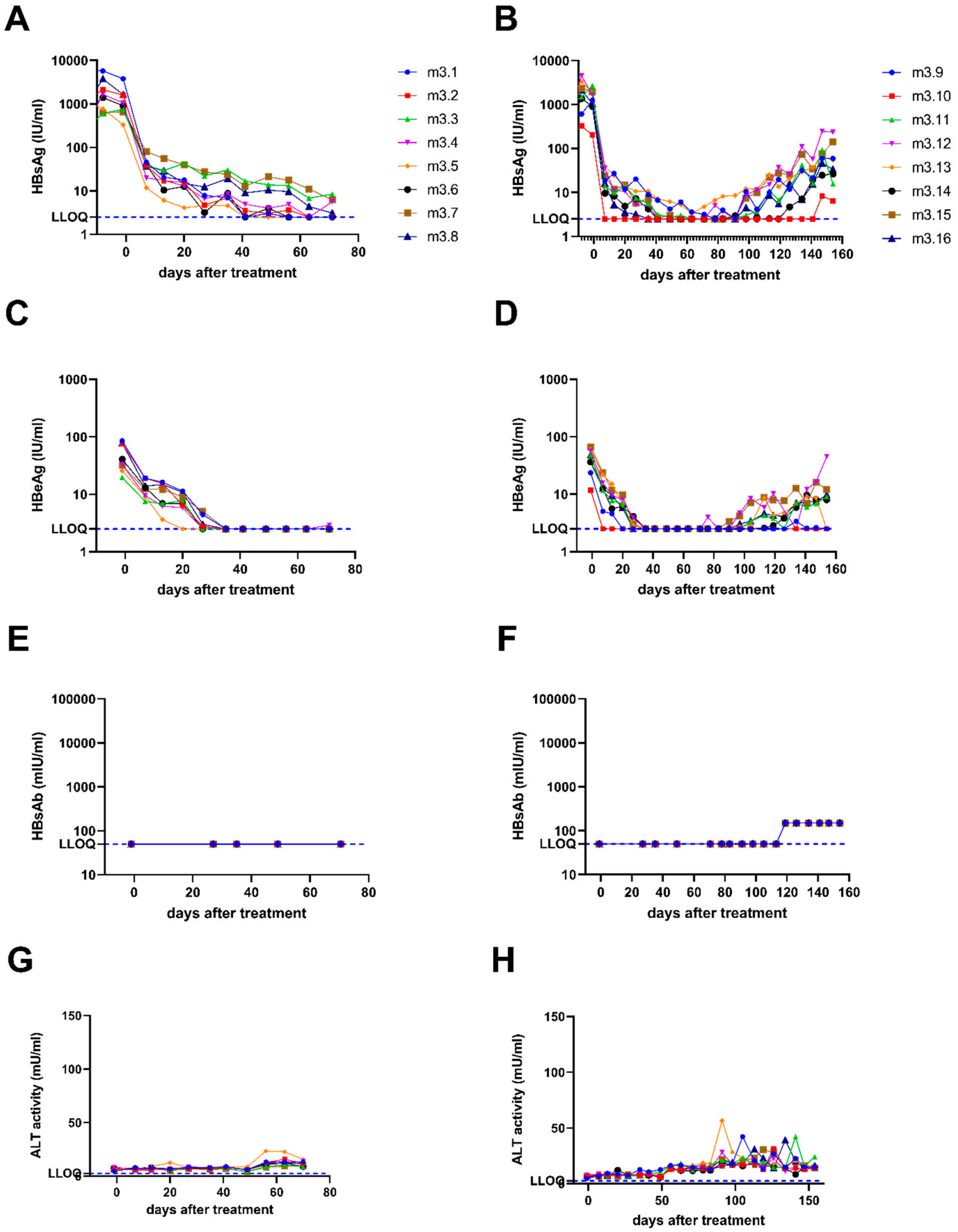

Figure S5- Single-cell RNA sequencing QC and annotation. AAV-HBV transduced C57BL/6 mice were treated with GalNAc-HBV siRNA and therapeutic vaccine (G1), with GalNAc-control siRNA and therapeutic vaccine (G2) and with GalNAc-control siRNA and mock vaccine (G3). Intrahepatic immune cells from liver were isolated 1 week after the administration of the second therapeutic vaccine (timepoint day 70) or 7 weeks after the 4th therapeutic vaccine (timepoint day 154). **(A)** Number of cells after QC per sample **(B)** Dendrogram of the levels of cell annotation and cell numbers.

a

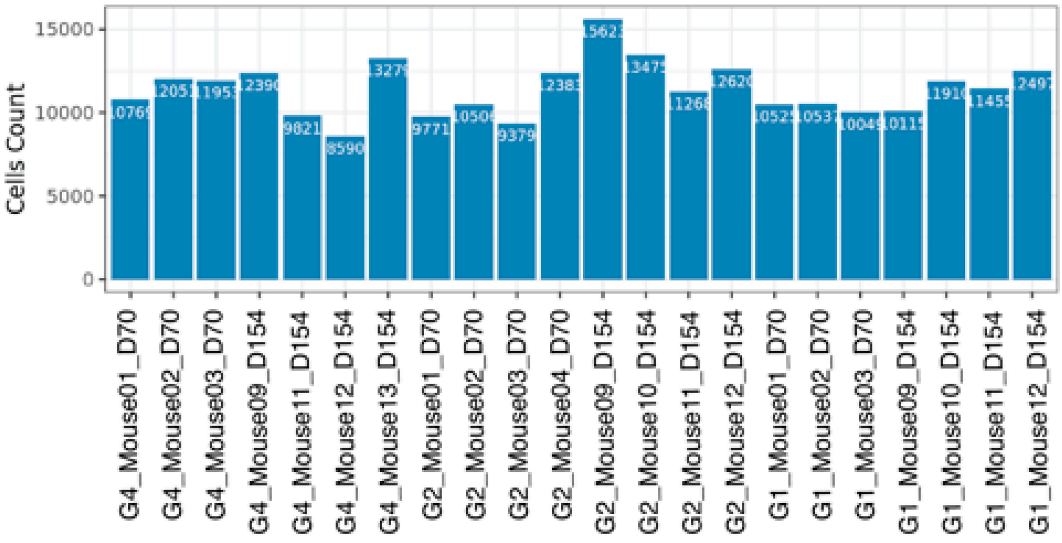

b

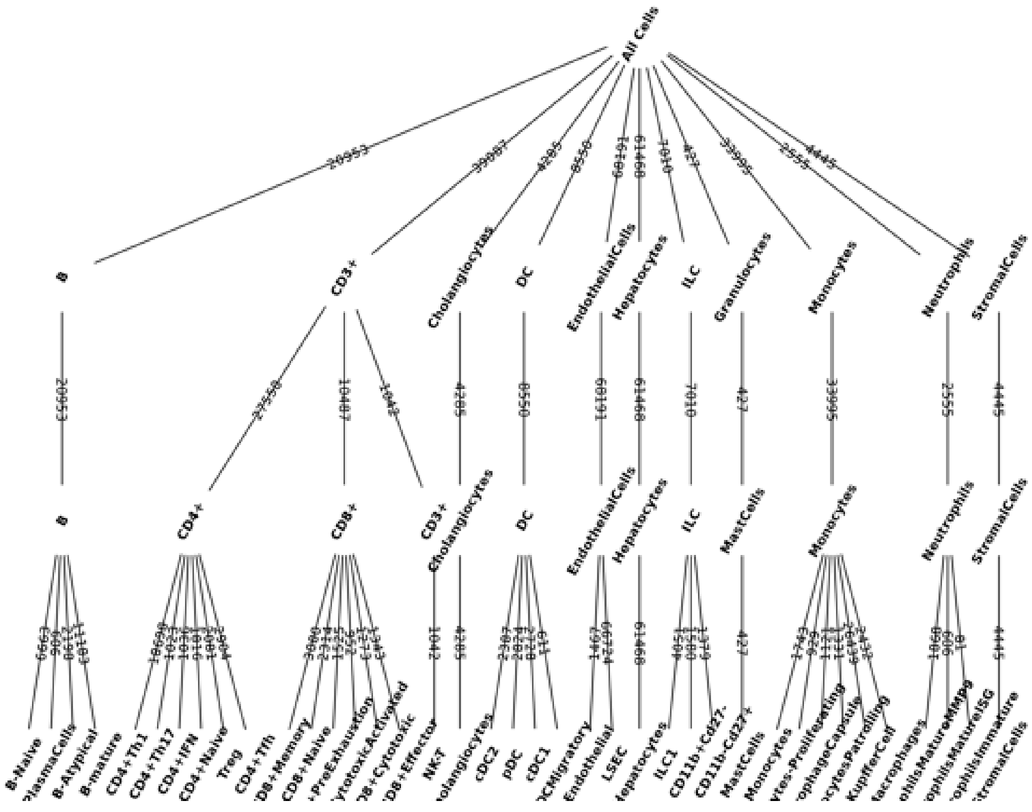

Figure S6 – CD8 T cell frequency and pre-exhaustion profiles. AAV-HBV transduced C57BL/6 mice were treated with GalNAc-HBV siRNA and therapeutic vaccine, with GalNAc-control siRNA and therapeutic vaccine and with GalNAc-control siRNA and mock vaccine. Intrahepatic immune cells from liver were isolated 1 week after the administration of the second therapeutic vaccine (timepoint day 70) or 7 weeks after the 4th therapeutic vaccine (timepoint day 154). **(A)** Frequency of CD8 T cells across groups and statistical comparison at day 154 (paired wilcox test, non-parametric) with fdr adjustment. CD8+Cytotoxic at day 154 from GalNAc-HBV siRNA and therapeutic vaccine vs mock control was significant (p=0.029, p.adj=0.087). CD8+PreExhaustion at day 154 from GalNAc-HBV siRNA and therapeutic vaccine vs mock control was significant (p=0.029, p.adj=0.0855). CD8+CentralMemory at day 154 from GalNAc-HBV siRNA and therapeutic vaccine vs mock control was significant (p=0.029, p.adj=0.0435) and from GalNAc-HBV siRNA and therapeutic vaccine vs GalNAc-control siRNA and therapeutic vaccine (p=0.029, p.adj=0.0435). CD8+CytotoxicActivated at day 154 from GalNAc-HBV siRNA and therapeutic vaccine vs G4 was significant (p=0.026, p.adj=0.039) and from GalNAc-HBV siRNA and therapeutic vaccine vs GalNAc-control siRNA and therapeutic vaccine (p=0.026, p.adj=0.039). **(B)** CD8 pre-exhaustion T cells dot plot of scaled expression of Tcf7 and Id3 across the different groups.

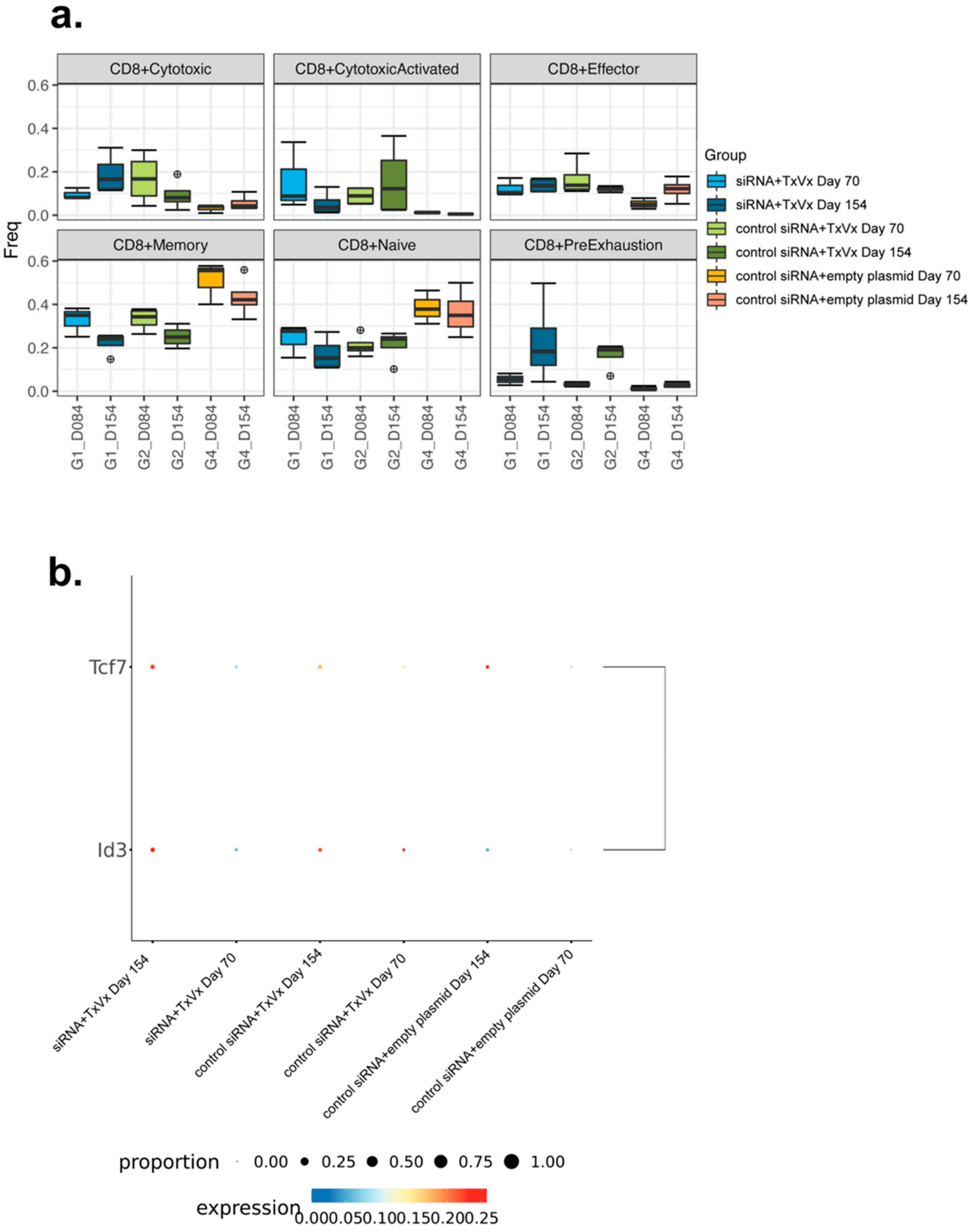

Figure S7 – Monocyte and DC compartment frequency analysis. AAV-HBV transduced C57BL/6 mice were treated with GalNAc-HBV siRNA and therapeutic vaccine, with GalNAc-control siRNA and therapeutic vaccine and with GalNAc-control siRNA and mock vaccine. Intrahepatic immune cells from liver were isolated 1 week after the administration of the second therapeutic vaccine (timepoint day 70) or 7 weeks after the 4th therapeutic vaccine (timepoint day 154). **(A)** Frequency of dendritic cells across groups and statistical comparison at day 154 (paired wilcox test, non-parametric, fdr adjustment). pDCs at day 154 from GalNAc-HBV siRNA and therapeutic vaccine vs GalNAc-control siRNA and therapeutic vaccine were significant (p=0.029, p.adj=0.087). Migratory DCs at day 154 from GalNAc-HBV siRNA and therapeutic vaccine vs mock control were significant (p=0.029, p.adj=0.057). **(B)** Frequency of Monocytes/Macrophages across groups and statistical comparison at day 154 (paired wilcox test, non-parametric, fdr adjustment). Macrophage Capsule at day 154 from GalNAc-HBV siRNA and therapeutic vaccine vs mock control was significant (p=0.029, p.adj=0.087).

a.

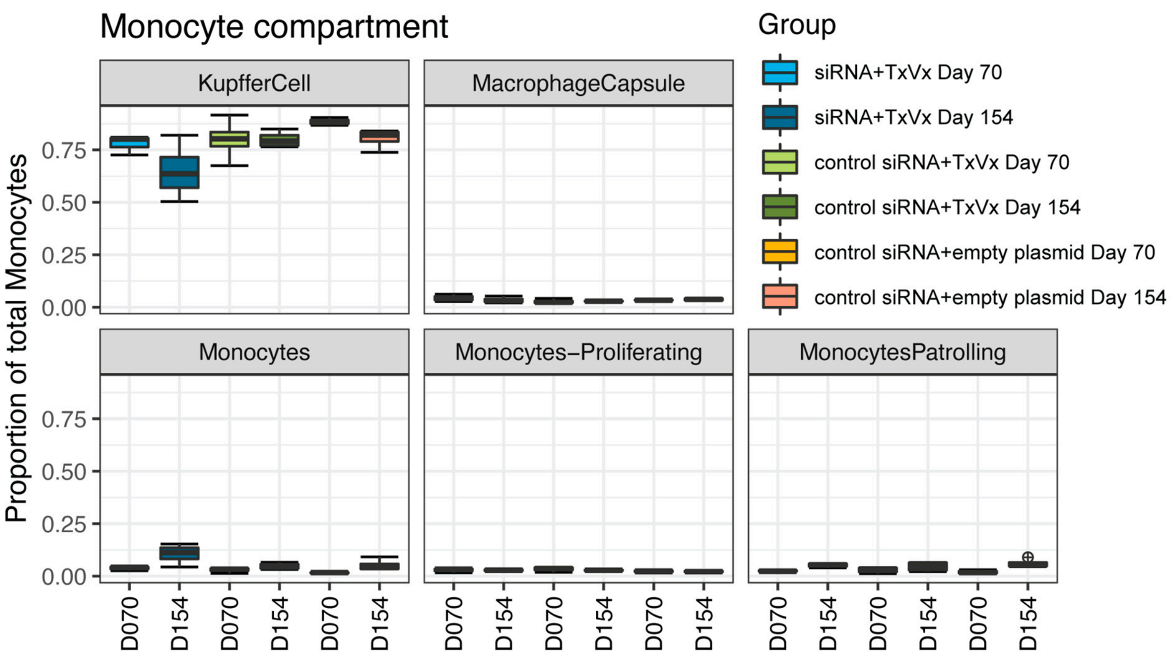

b.

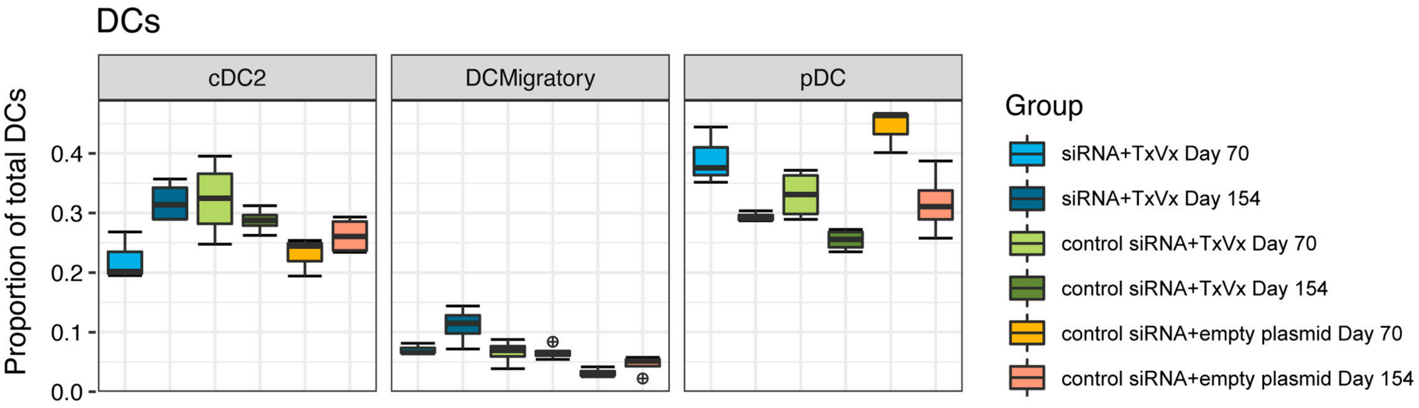

Figure S8– Neutrophil compartment analysis. AAV-HBV transduced C57BL/6 mice were treated with GalNAc-HBV siRNA and therapeutic vaccine, with GalNAc-control siRNA and therapeutic vaccine and with GalNAc-control siRNA and mock vaccine. Intrahepatic immune cells from liver were isolated 1 week after the administration of the second therapeutic vaccine (timepoint day 70) or 7 weeks after the 4th therapeutic vaccine (timepoint day 154). **(a)** Dotplot of scaled expression of hallmark genes in the neutrophil compartment. **(b)** Violin plots of normalized expression of IFN/activation genes (*Isg15, Cd274, Rsad2*) and immunosuppressive genes (*Arg1, Mmp9, Mmp8*)

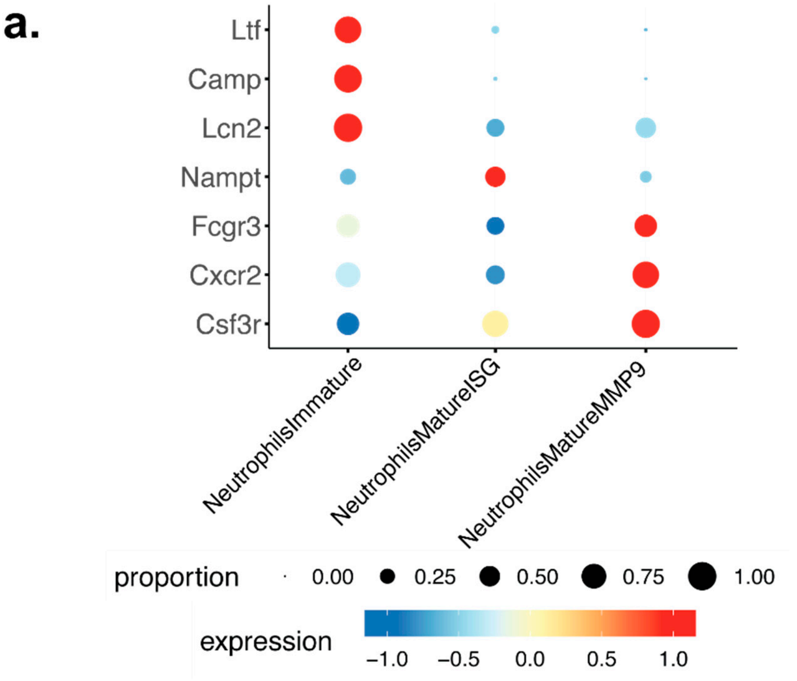

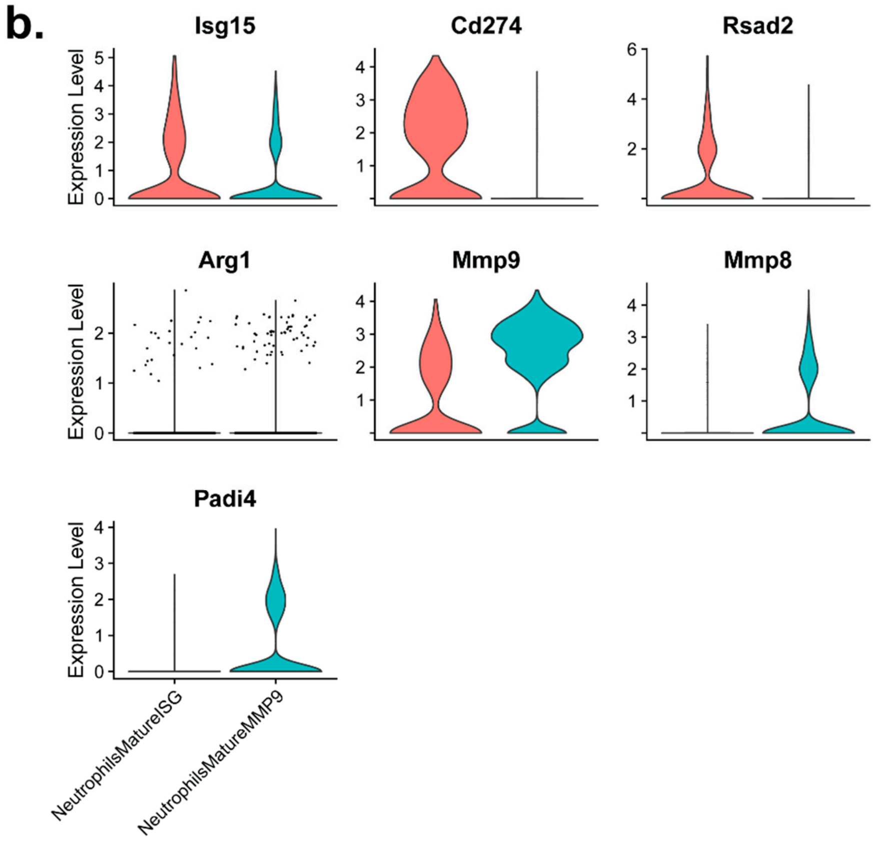

Figure S9 – ILC compartment frequency analysis. AAV-HBV transduced C57BL/6 mice were treated with GalNAc-HBV siRNA and therapeutic vaccine, with GalNAc-control siRNA and therapeutic vaccine and with GalNAc-control siRNA and mock vaccine. Intrahepatic immune cells from liver were isolated 1 week after the administration of the second therapeutic vaccine (timepoint day 70) or 7 weeks after the 4th therapeutic vaccine (timepoint day 154). **(A)** Frequency of ILCs across groups and statistical comparison at day 154 (paired wilcox test, non-parametric) with fdr adjustment. NK_CD11b+Cd27-at day 154 from GalNAc-HBV siRNA and therapeutic vaccine vs mock control were significant (p=0.029, p.adj=0.087). **(B)** Dot plot with scaled expression of hallmark genes in the ILC compartment.

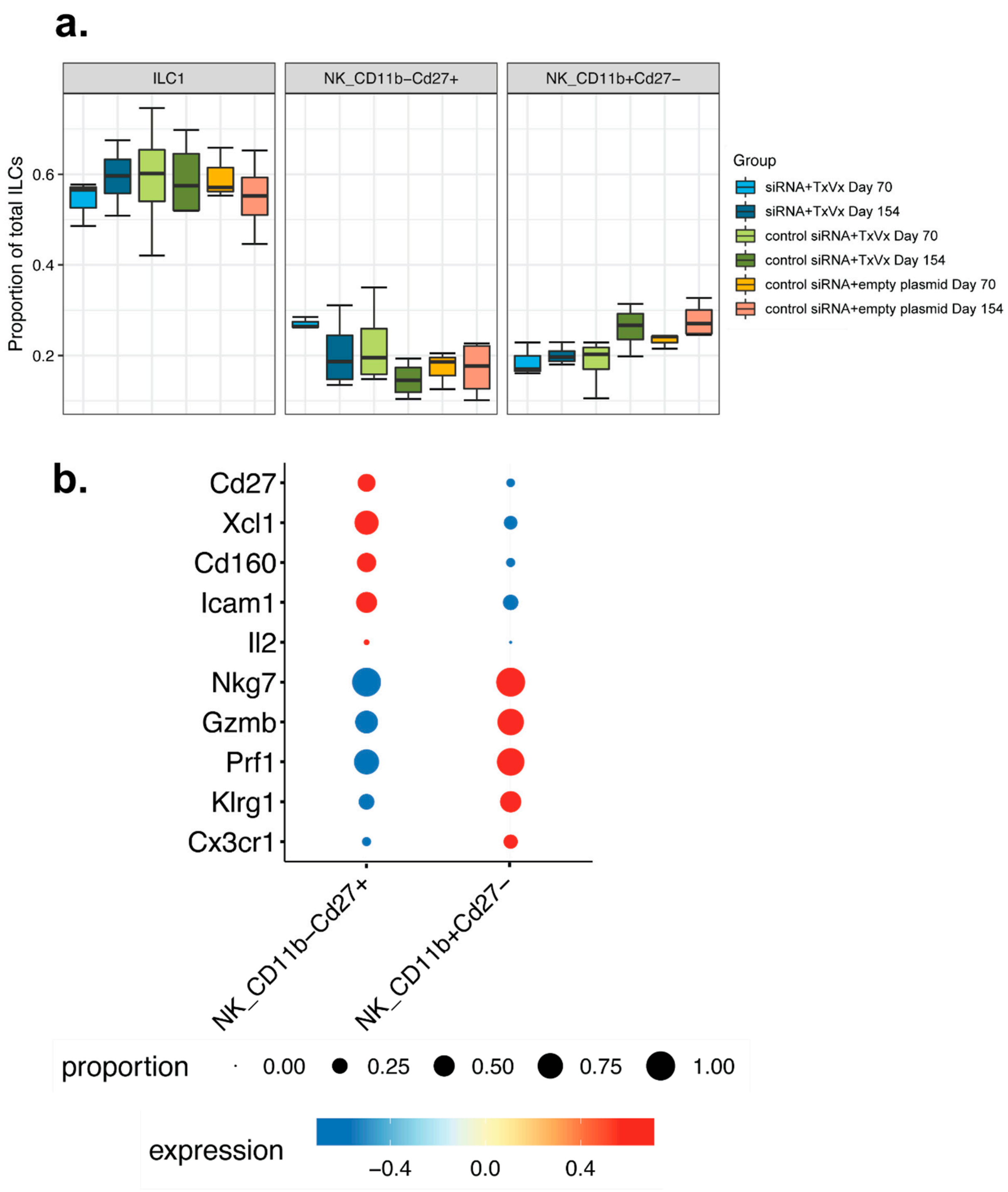

Figure S10 –TCR clonality frequency AAV-HBV transduced C57BL/6 mice were treated with GalNAc-HBV siRNA and therapeutic vaccine (G1), with GalNAc-control siRNA and therapeutic vaccine (G2) and with GalNAc-control siRNA and mock vaccine (G4). Intrahepatic immune cells from liver were isolated 1 week after the administration of the second therapeutic vaccine (timepoint day 70) or 7 weeks after the 4th therapeutic vaccine (timepoint day 154). **(A)** Frequency of the TCR clonal expansion in the CD4 compartment per group **(B)** Frequency of the TCR clonal expansion in the CD8 compartment per group.

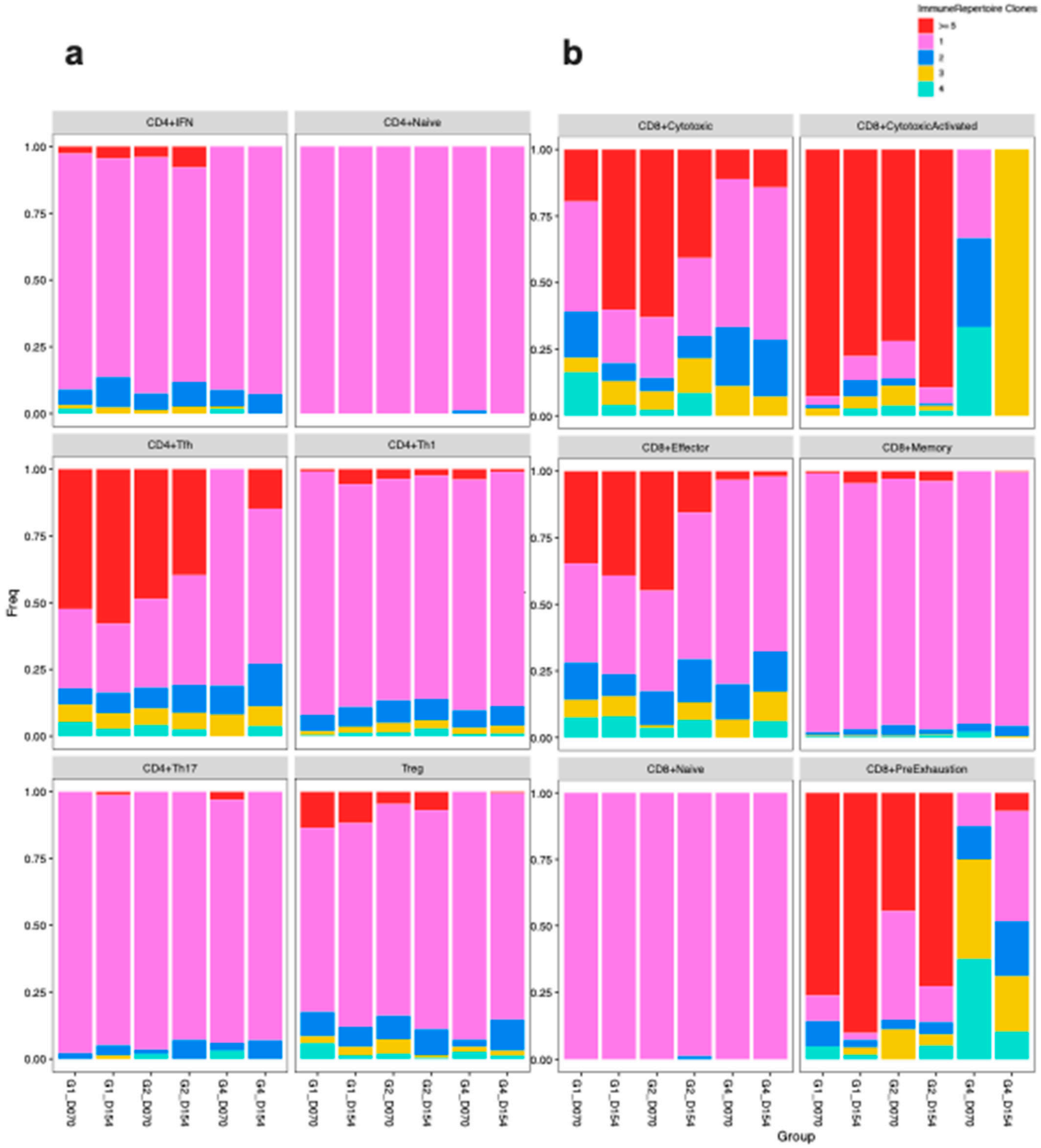

Figure S11 – B cell compartment analysis. AAV-HBV transduced C57BL/6 mice were treated with GalNAc-HBV siRNA and therapeutic vaccine (G1), with GalNAc-control siRNA and therapeutic vaccine (G2) and with GalNAc-control siRNA and mock vaccine (G4). Intrahepatic immune cells from liver were isolated 1 week after the administration of the second therapeutic vaccine (timepoint day 70) or 7 weeks after the 4th therapeutic vaccine (timepoint day 154). **(A)** Violin plots of normalized expression from B-cell marker genes for each cell subtype **(B)** Frequency of the BCR clonal expansion in each B cell subtype, in red are depicted the expanded clonotypes. **(C)** Dotplot of scaled expression of Long-Lived Plasma Cell marker genes in Plasma cells, across clonality groups, >=3 clonotypes are expanded. **(D)** Phylogeny analysis from B cell clones (CDR3 sequences) of samples from the siRNA and therapeutic vaccine combination **(E)** from the control siRNA and therapeutic vaccine treatment **(F)** and from the control siRNA and empty plasmid.

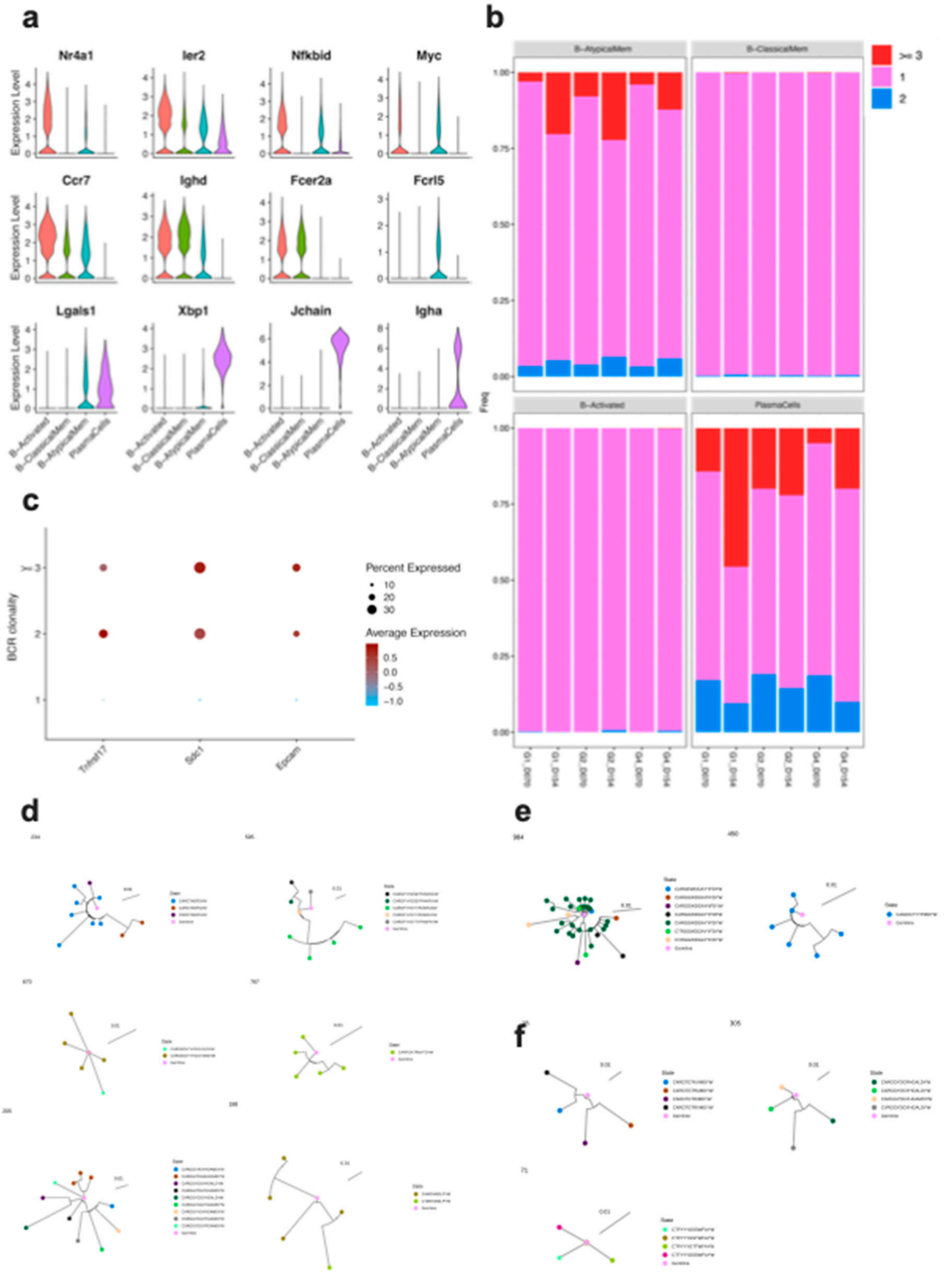

Supplementary Table S1 – Percentage distribution of cell types

CellType	TotalCells	Percentage
B	20953	8
CD3+	39087	16
Cholangiocytes	4285	2
DC	8550	3
EndothelialCells	68191	27
Granulocytes	427	0
Hepatocytes	61468	24
ILC	7010	3
Monocytes	33995	14
Neutrophils	2555	1
StromalCells	4445	2
Totals	250966	100

Supplementary Table S2 – Frequency distribution of clonal expansion in CD8 total T cells

Group	Timepoint	CellType	Clonality	Proportion
siRNA+TxVx	D070	Cd8	>= 5	32.82
siRNA+TxVx	D154	Cd8	>= 5	53.31
controlsiRNA+TxVX	D070	Cd8	>= 5	30.87
controlsiRNA+TxVX	D154	Cd8	>= 5	38.75
controlsiRNA+empty plasmid	D070	Cd8	>= 5	0.72
controlsiRNA+empty plasmid	D154	Cd8	>= 5	2.02

Supplementary Table S3 – Frequency distribution of clonal expansion in CD4 total T cells

Group	Timepoint	CellType	Clonality	Percentage
siRNA+TxVx	D070	Cd4	>= 5	7.94
siRNA+TxVx	D154	Cd4	>= 5	23.28
controlsiRNA+TxVX	D070	Cd4	>= 5	9.17
controlsiRNA+TxVX	D154	Cd4	>= 5	9.80
controlsiRNA+empty plasmid	D070	Cd4	>= 5	3.40
controlsiRNA+empty plasmid	D154	Cd4	>= 5	1.51
